# Collecting and using social needs data in health settings: a systematic review of the literature on health service utilization and costs

**DOI:** 10.1186/s12913-025-13458-2

**Published:** 2025-09-30

**Authors:** Mélanie Ann Smithman, Oluwasegun J. Ogundele, Laure Perrier, Menna Komeiha, Iryna Artyukh, Paras Kapoor, Andrew D. Pinto

**Affiliations:** 1 Upstream Lab, MAP Centre for Urban Health Solutions, Li Ka Shing Knowledge Institute, Unity Health Toronto, 30 Bond Street, Toronto, Ontario M5B 1W8 Canada; 2 Université de Sherbrooke, Quebec, Sherbrooke, Canada; 3 Ontario Hospital Association, Toronto, Ontario Canada; 4 Institute for Health Policy, Management and Evaluation, Dalla Lana School of Public Health, University of Toronto, Toronto, Ontario Canada; 5 Department of Family and Community Medicine, Faculty of Medicine, University of Toronto, Toronto, Ontario Canada; 6 Department of Family and Community Medicine, St. Michael’s Hospital, Unity Health Toronto, Toronto, Ontario Canada

**Keywords:** Social determinants of health, Healthcare costs, Health service utilization, Systematic review, Emergency department, Hospitalization, Health inequities, Social screening

## Abstract

**Background:**

Social determinants of health significantly influence health outcomes and contribute to health inequities across populations. Systematic and routine collection of social needs data and its use to inform interventions within healthcare settings are proposed to reduce health services utilization and healthcare costs. This systematic review examines the impact of social needs data collection and use on health service utilization and healthcare costs.

**Methods:**

Following PRISMA guidelines, we conducted a systematic review of studies published between January 2015 and February 2024. We included studies that reported on the collection and use of social needs data within healthcare settings in high-income countries. The review included randomized controlled trials, observational studies, quasi-experimental studies, qualitative studies, quality improvement studies, and mixed methods designs. Databases searched included Ovid MEDLINE, EMBASE, and Cochrane CENTRAL. The primary outcomes assessed were changes in health service utilization and healthcare costs.

**Results:**

The review identified 35 relevant studies, predominantly from the United States. Interventions utilizing social needs data were implemented across various healthcare settings, including emergency departments, primary care, and inpatient facilities. Most studies reported reductions in emergency department visits (13/35) and hospitalizations (14/35) associated with collecting and using social needs data. Several studies demonstrated associated cost reductions, particularly in emergency department and hospitalization costs. However, the findings were mixed, with some studies reporting no significant changes or increased costs in certain areas, such as diagnostic testing and ambulatory care.

**Conclusions:**

Collecting and using social needs data within healthcare settings shows potential for reducing health service utilization and associated costs, particularly in targeted populations. The variability in outcomes suggests the need for context-specific approaches and further research to standardize reporting and understand the long-term impacts of these interventions. Standardized reporting and more robust study designs are needed to better understand these interventions’ long-term impact, best implementation strategies, and scalability.

**Supplementary Information:**

The online version contains supplementary material available at 10.1186/s12913-025-13458-2.

## Background

The social determinants of health [1] significantly impact health outcomes and health inequities [2, 3]. Over the past decade, an increasing number of healthcare settings have implemented approaches to collect data on patients’ social needs, such as socioeconomic status, educational attainment, housing status, food security, access to transportation, etc. Social needs data can be used to improve patient care by integrating social needs-informed interventions into healthcare delivery, developing new programs or services, and addressing inequities in healthcare access and outcomes at the individual, practice, or population level [4–6].

Social needs – particularly those related to low income, low educational attainment, and food insecurity – are associated with higher health service utilization and costs [7, 8]. Patients experiencing high social needs may have less access to preventive and primary care, may delay seeking care in part due to past experiences of discrimination, may have more health needs, and have longer hospital stays. Collecting and using data to address patients’ social needs could help decrease health services utilization and healthcare costs [9]. Data could help build the rationale for early interventions that meet social needs and improve health over the medium- to long-term. Social needs data could be used to risk-adjust payments to health professionals, incentivizing them to provide services to patients made vulnerable by socioeconomic inequities. Social needs data could also predict hospitalizations or hospital readmission, helping teams engage in proactive outreach to specific groups.

Previous reviews have highlighted the growing integration of interventions to address social needs in healthcare settings [10–12]. However, the evidence base remains limited, focusing either on social screening or social interventions in specific settings or groups, with heterogeneous reporting, varied outcome measures, and a predominant focus on process and social outcomes rather than health and healthcare outcomes. A recent review by Yan et al. examined the effectiveness of integrating social needs screening into electronic health records in the United States, finding mixed but generally positive impacts on healthcare utilization and costs [11]. Building on that work, we examined how social needs data collection—whether captured via electronic health records integration or other methods—affects healthcare utilization and costs in high-income countries. We focus on high-income countries to ensure comparability among well-resourced systems equipped for systematic interventions and a broader view of how different healthcare systems integrate and use social needs data. We address the following question: *What are the impacts of social needs data collection and use on health service utilization and healthcare costs?*

## Methods

This systematic review followed the Preferred Reporting Items for Systematic Reviews and Meta-Analyses Extension for Systematic Reviews (PRISMA) guidelines [13].

### Eligibility criteria

The population (P) of interest in this review comprised individuals and communities in high-income countries within healthcare settings. The interventions (I) involved the systematic collection and use of social needs data, particularly initiatives such as screening for social needs and tailoring healthcare interventions based on factors like education, income, housing, social isolation, transportation, utilities, and employment. Comparators (C) included healthcare settings or groups that had not received such interventions, or periods before and after the implementation of social needs data collection and interventions informed by this data. The outcomes (O) of interest were health service utilization and healthcare costs. The inclusion and exclusion criteria are described in Table 1.

**Table 1:** Eligibility criteria

Inclusion	Exclusion
Intervention: studies describing interventions addressing collected social needs data (e.g., poverty, socioeconomic status, food security)	• Interventions without social needs data collection or social needs screening • Studies that did not address identified social needs through an explicit intervention
Setting: healthcare settings (e.g., emergency department visits, hospitalizations, primary care visits) AND in high-income countries	Low-income settings, non-healthcare settings
Study period: January 2015 to February 2024	Other periods
Language: English	Non-English
Reporting on the impact on: • health service utilization (e.g., clinic visits, ED visits, hospitalization) AND/OR • costs of healthcare services (e.g., total health care costs, societal costs, out-of-pocket expenses, utilization patterns and costs)	Not reporting impacts on these outcomes
Study design: quantitative, qualitative, quality improvement, mixed methods	Study design: in vitro, systematic reviews
Sources: peer reviewed articles, conference abstracts	Sources: theses, dissertations, grey literature, research protocols, commentaries, editorials, correspondences, preprint articles or no original data reported

### Search strategy

A comprehensive literature search was run in Ovid MEDLINE, Ovid MEDLINE Daily, Ovid MEDLINE In-Process & Other Non-Indexed Citations, Ovid MEDLINE Epub Ahead of Print, OVID EMBASE, and Cochrane CENTRAL. The search strategy was designed and executed by an experienced information specialist (LP) with input from the co-authors (MAS, OJO, and AP). Searches were performed from January 2015 to February 2024 and limited to English language only. Detailed search strategies can be found in supplementary file A. All search results were imported into Covidence (covidence.org) for reference management.

### Additional search strategy

The references in systematic reviews were screened to find relevant primary studies. Additionally, AI-assisted snowballing was employed to identify further relevant articles by analyzing selected pertinent reviews. Investigators provided articles deemed eligible and relevant, and several others were identified in the comprehensive literature search. The research question was submitted to Consensus (https://consensus.app/search/) to generate additional relevant articles. A dozen articles were then submitted to Research Rabbit (https://www.researchrabbit.ai/) to generate related publications for screening. All related publications generated by Research Rabbit were screened by one team member (MAS). Those meeting eligibility criteria based on their titles and abstracts were included for full-text assessment.

### Study selection

Titles and abstracts identified through the search were assessed against a predefined checklist to exclude irrelevant citations. Five reviewers independently screened 25 records to calibrate the process, followed by additional training (MK, IA, PK, MAS, OJO). Once consistency was confirmed (90% agreement), the remaining records were independently screened by at least two reviewers.

Titles and abstracts identified in the search were independently screened by the review team (MK, IA, PK, NS, LM, USR, HS, MAS, OJO) to exclude publications that were irrelevant based on the following inclusion and exclusion criteria:

Full texts were obtained for all titles that remained after the initial screening. Full-text articles were read, and those that did not satisfy the inclusion criteria were subsequently removed (see Table 1 for criteria). Full-text copies of potentially eligible studies were reviewed independently by at least two members of the review team to ensure they met the inclusion criteria, with discrepancies resolved by a third reviewer (MAS). All final papers were assessed by a third and fourth reviewer (MAS and OJO) to minimize the risk of inclusion bias.

### Data collection process and items

Data were collected using a form (developed by MAS and OJO) to extract data—such as study design, setting, sample characteristics, intervention details, outcomes, results, and key discussion points. The data extraction form was pilot tested on three articles and modified accordingly. Four members of the review team independently extracted relevant information from the articles, and reviewed by one team member (MAS). All authors reviewed and discussed each step of the data extraction process and the related outputs to address any questions or uncertainties.

### Quality assessment

Quality appraisal of studies was assessed using the Mixed Methods Appraisal Tool (MMAT, 2018 Version) [14]. The MMAT allows for evaluating studies of various designs, including qualitative research, randomized controlled trials, non-randomized studies, and quantitative descriptive studies, and has been pilot tested for efficiency, content validity, and reliability. Each criterion is assessed as either met (‘Yes’), not met (‘No’) or insufficient information for judgment (‘Can’t tell’). The quality of studies was not a criterion for exclusion but was used to inform support recommendations for improving reporting or rigour in future studies. Quality assessment was completed independently by the review team (MK, IA, PK) and confirmed by a second reviewer (OJO).

### Data synthesis

The findings were synthesized into tables and summarized narratively, integrating both quantitative and qualitative data. The synthesis included specific details of the methods for collecting and analyzing social data, as well as the interventions and outcomes of interest. Qualitative content analysis, as described by Elo and Kyngäs [15], was used for data synthesis. A key advantage of content analysis is its flexibility in research design, allowing for both deductive and inductive approaches depending on the research objectives [15].

## Results

**Fig. 1:**
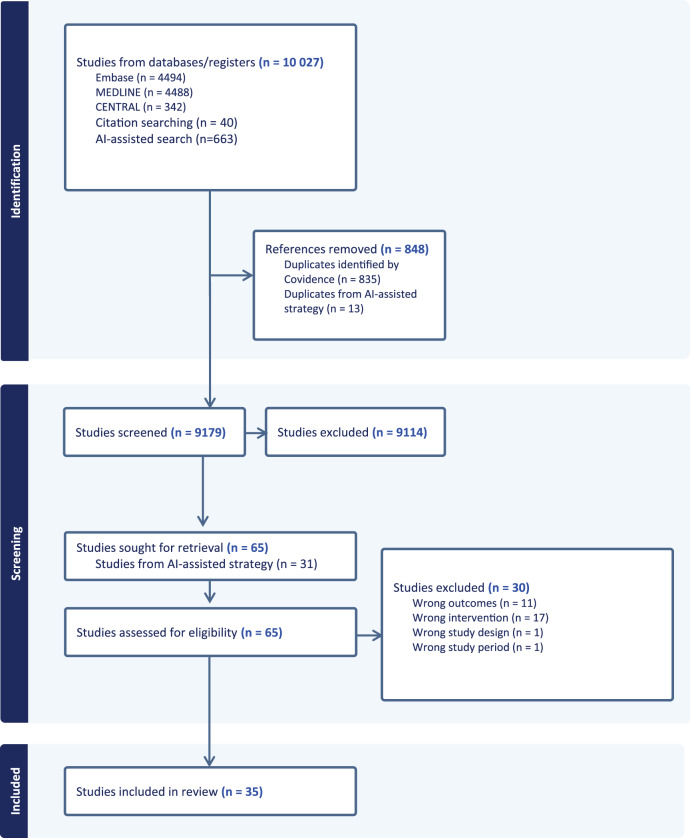
PRISMA study flow diagram

A total of 10,027 studies were identified (see Fig. 1). Database searches yielded 9324 studies (Embase *n* = 4,494; MEDLINE *n* = 4,488; CENTRAL *n* = 342). Citation screening from systematic reviews added 40 articles. A dozen relevant articles from the database searches and citation screening were used as a basis for the AI-assisted search, which added 663 records. After removing 848 duplicates, 9,179 titles and abstracts were screened, resulting in 9,114 exclusions. Sixty-five full-text articles were assessed for eligibility, of which 31 had been identified through the AI-assisted search strategy. In total, 35 studies met the inclusion criteria and were included in this systematic review.

Characteristics of the included studies are presented in Table 2. The large majority (32/35) of studies were conducted in the United States [16, 18–28, 30–47, 49, 50] one in Canada [17], and two in the United Kingdom [29, 48]. Most studies had experimental designs (17/35) [16, 18–46, 49, 50], nine were observational [19–28, 30–39, 41–47, 49, 50], five were quasi-experimental [30, 32–37, 39, 48, 50], and four were mixed-methods [18–28, 30–43, 46, 49, 50].

**Table 2:** Characteristics of the included studies

Author (year)	Study setting	Study aim	Target population	Sample size
Berkowitz et al. (2018) [16]	Members of the Commonwealth Care Alliance, involved a ‘Meals on Wheels’ program (meals delivered to the participant’s home), USA	To determine whether home delivery of either medically tailored meals (MTM) or non-tailored food (NTF) reduces healthcare utilization and expenditure	Adults (age > 21 years) who are dually eligible for Medicaid and Medicare	3 077
Bradley et al. (2018) [17]	Four health professions schools at an academic health science university and a partner state university, Portland, Oregon, USA	To describe the development, interventions and evaluation of the I-CAN model which aims to reduce preventable health-care utilization among underserved clients and families in partner neighborhoods by addressing the social determinants of health, partnering with communities and preparing an interprofessional health-care workforce	Patients that have a lack of a primary care home, lack of health insurance, miss medical appointments, and/or lack of stable housing. Specific target populations: Site 1 – chronically sub-acute; elderly; homeless, disabled; veterans. Site 2 – Single parent families; disabled adults, Hispanic migrant works. Site 3 – Immigrants and refugees from Africa and Asia	38
Bronstein et al. (2015) [18]	A nonprofit regional hospital in upstate New York, USA	To test the impact of a social worker-led care coordination intervention administered by interns enrolled in Master of social work studies on within-30-day readmission rates	Adults 50 or older at moderate or high risk of rehospitalization	89
Capp et al. (2017) [19]	A large urban academic medical center (in a low income community) and another local hospital, Colorado., USA	To evaluate how participation in Bridges to Care (B2C) an ED-initiated, multidisciplinary, community-based program affected subsequent ED use, hospital admissions, and primary care use	Publicly insured or Medicaid-eligible high utilizers of acute (ED and inpatient) care	3 802
DeLaVega et al. (2022) [20]	A general internal medicine practice at a large safety-net hospital in Boston, Massachusetts, USA	To evaluate an enhancement of pharmacy care to reduce hospital admissions and ED visits among primary care patients in a Medicaid accountable care organization	Primary care patients in a Medicaid accountable care organization	364
Finkelstein et al. (2020) [21]	Four Camden-area hospital systems, New Jersey, USA	To evaluate a nationally recognized program: the Camden Core Model	Super utilizers of the health care system persons with medically and socially complex needs who have frequent hospital admissions	782
Gupta et al. (2023) [22]	Largest private non-profit health System in South Carolina (Midlands and Northwestern Upstate regions), USA	To assess the feasibility and sustainability of social determinants of health screening and related referrals in a large health system and examine how they affect health resource use	Prisma Health patients aged 18 years and over	2 723
Haggerty et al. (2023) [23]	Monteregie, Quebec. Family doctor’s clinic, Canada	To evaluate the extent to which the volunteer navigator outreach helped patients reach and engage with their newly-assigned primary care team, have a positive healthcare experience, develop an enduring doctor-patient relationship, and reduce forgone care and ED use	Unattached persons who did not have a family doctor from deprived neighborhoods	60
Heisler et al. (2022) [24]	Cody Rouge, neighborhood, Detroit, Michigan, USA	To compare health care utilization and costs between beneficiaries randomly assigned to usual services versus a community health worker (CHW) program	Patients enrolled in one of 3 Medicaid health plans with a least 3 ED visits or 1 ambulatory care sensitive hospitalization in the previous 12 months	2 457
Henschen et al. (2022) [25]	Northwestern Memorial Hospital, a large, urban academic hospital that serves a diverse patient population in Chicago, Illinois, USA	To compare hospital utilization metrics among patients enrolled in Complex High Admission Management program (CHAMP) and usual care	Frequently Hospitalized Patients	151
Kangovi et al. (2017) [26]	2 academic Philadelphia, Pennsylvania, adult internal medicine clinics, USA	To determine whether a community health worker (CHW) intervention - Individualized Management for Patient-Centered Targets (IMPaCT) - improved outcomes in a low-income population with multiple chronic conditions	High-poverty neighborhood residents, uninsured or publicly insured, and diagnosed with 2 or more chronic diseases (diabetes, obesity, tobacco dependence, hypertension)	302
Kangovi et al. (2018) [27]	3 primary care facilities in Philadelphia, Pennsylvania, USA	To assess a standardized intervention, Individualized Management for Patient-Centered Targets (IMPaCT), delivered by community health workers (CHWs)	Patients who resided in a high-poverty zip code, were uninsured or publicly insured, and who had a diagnosis for 2 or more chronic diseases (diabetes, obesity, tobacco dependence, hypertension)	592
Kangovi et al. (2020) [28]	2 academic Philadelphia, Pennsylvania, adult internal medicine clinics, USA	To calculate a return on investment of a community health worker (CHW) program, Individualized Management for Patient-Centered Targets (IMPaCT), from the perspective of a Medicaid payer	High-poverty neighborhood residents, uninsured or publicly insured, and diagnosed with 2 or more chronic diseases (diabetes, obesity, tobacco dependence, hypertension)	302
Kenyon et al. (2016) [29]	3 Maternity Trusts in West Midlands, United Kingdom	To evaluation effectiveness of lay support to improve maternal and child outcomes in disadvantaged families	Nulliparous women under 28 weeks gestation, with social risk factors	1 324
Kitzman et al. (2022) [30]	Community-based Patient Centered Medical Home: Baylor Scott & White Health and Wellness Center, USA	To evaluate the impact of population health strategies focused on social determinants of health and integrated into the primary care medical home, on diabetes and cardiovascular disease outcomes We also investigated associations between these outcomes and emergency department (ED) and inpatient (IP) use and costs	Low-income, primarily ethnic minority community patients who attended at least two primary care visits within a 12-month time span	445
Liss et al. (2019) [31]	Northwestern Memorial Hospital in Chicago, Illinois, USA	To evaluate effects of a transitional care practice that comprehensively addresses patients’ medical and psychosocial needs following hospital discharge	Adults discharged from an initial emergency, observation, or inpatient hospital encounter with no trusted usual source of care	654
Losonczy et al. (2017) [32]	A safety net county-owned hospital, USA	To determine the social needs of patients presenting to the ED and evaluate the impact of the Highland Health Advocates (HHA) in resolving those needs and connecting them to a ‘medical home’	Adult ED patients at a safety net county-owned hospital	459
Moreno et al. (2021) [33]	Community-based program in Southern California, USA	To examine the impact of the Connecting Provider to Home program on utilization and satisfaction with care	Older adult patients with multiple medical conditions and complex social issues	420
Onwuanyi et al. (2020) [34]	A safety net hospital, USA	To identify, incorporate and manage social determinants of health domains in chronic heart failure patients treated in a safety net hospital	Chronic heart failure patients	147
OToole et al. (2016) [35]	33 Veteran Health Administration facilities with homeless medical homes and patient- aligned care teams that served more than 14,000 patients, USA	To describe the national implementation of a homeless medical home initiative the Homeless Patient Aligned Care Team (H-PACT) and correlate patient health outcomes with characteristics of high-performing sites	Homeless veterans	3 543
Pantell et al. (2020) [36]	Primary and urgent care clinics of 2 safety-net health systems in northern California, USA	To compare the acute care utilization effects of a written resources handout vs an in-person navigation service intervention to address social needs	Adult caregivers of pediatric patients seen in primary and urgent care clinics	1 300
Pruitt et al. (2018) [37]	14 states - a managed care organizations call center-based social service referral program, USA	To evaluate the savings associated with a managed care organizations call center-based social service referral program that aimed to assist participants address their social needs beyond the typical 2–1–1 service offerings	Patients insured through Medicare Advantage or Medicaid managed care	2 718
Roth et al. (2023) [38]	Seven Providence clinics (three treatment and four control) in the tri-county area of Portland, Oregon, USA	To assess the impact of the Providence Diabetes Collective Impact Initiative (DCII), a multifaceted intervention approach to diabetes treatment that employed both clinical and social determinants of health strategies, on access to medical and social services	People aged 18–65 years old with a pre-existing type 2 diabetes diagnosis	1 220
Rucker et al. (2023) [39]	Two EDs affiliated with a free-standing, tertiary care, urban academic children’s hospital, Washington, DC, USA	To evaluate the effect of social needs navigation for adolescents on subsequent ED visits and community resource use and to identify characteristics associated with elevated social risk	Adolescents in the emergency department with unmet social needs	384
Schickedanz et al. (2019) [40]	Large integrated health care delivery system, Kaiser Permanente Southern California, USA	To evaluate impacts of a social needs screening and navigation program for adult predicted high utilizers on total medical visit utilization	Adult patients predicted to be in the top 1% of health care utilization in Kaiser Permanente Southern California	34 225
Schumacher et al. (2017) [41]	Two EDs in tertiary referral centers serving diverse population with various payers, USA	To see if an ED-initiated coaching intervention can increase patient engagement	Older, chronically ill patients with limited health literacy insured by Medicare scheduled for ED discharge	69
Sege et al. (2015) [42]	Pediatric primary care clinic at a major urban safety-net teaching hospital, USA	To test the hypothesis that the addition of a trained family specialist would be able to both support families and facilitate measurable improvements in health care quality	Families of healthy newborns (younger than 10 weeks of age)	330
Smith et al. (2021) [43]	ED at Bronson Methodist Hospital in Michigan, with intervention being implemented in a community setting, USA	To see if a collaborative partnership between hospital and community resources can decrease nonemergent ED visits and associated costs by providing stable housing and better coordinated mental and physical health support for ED frequent user patients	Frequent ED users from hospital, experiencing homelessness and chronic pain	11
Vasan et al. (2020) [44]	Three urban, academically affiliated adult internal medicine primary care practices: a Veterans Affairs primary care practice, a federally qualified health center, and an academic family practice clinic in high-poverty regions of Philadelphia, USA	To analyze the effects of a standardized community health worker (CHW) intervention on hospitalization	Hospitalized patients who were publicly insured or uninsured	1 340
Vest et al. (2018) [45]	Nine federally qualified health centers operated by Eskenazi Health in Indianapolis, Indiana, USA	To measure the association between receiving wraparound services and patient outcomes in an eleven-year panel of adult patients	Adults aged 18 and older	14 094
Wallace et al. (2020) [46]	A large academic center ED, Utah, USA	To apply an evidence-based process-improvement model to develop a clear social needs assessment, referral, and evaluation process, to examine its feasibility during routine care delivery in the ED; and to examine the nature, quality, and usefulness of associating data from the social needs assessment, a database of community- based service referrals, and select fields from electronic health records	Patients in the ED	162
Weerahandi et al. (2015) [47]	Mount Sinai Medical Center in East Harlem, New York City, USA	To investigate whether the social work-led Preventable Admissions Care Team program (PACT) is effective in reducing the rate of 30-day readmissions, inpatient utilization, and costs	Patients aged 18 and older with one admission in 30 days or two hospitalizations in six months prior	538
Woodhead et al. (2017) [48]	Eight general practice sites in North London, England, United Kingdom	To examine the impact and cost-consequences of co-located benefits and debt advice on mental health and service use	Patients aged 18 and older	901
Wu et al. (2019) [49]	Johns Hopkins Hospital, Johns Hopkins Bayview Medical Center, and Johns Hopkins Medicine clinic sites, USA	To reduce healthcare utilization and increase the referral of patients between an academic health center and local community-based organizations that address social determinants of health with the Johns Hopkins Community Health Partnership (J-CHiP), a large-scale quality improvement project	Medical patients aged 18 and older with a least one chronic condition who were at high risk for future hospitalization	5255 patients, 22 community-based organizations
Xiang et al. (2019) [50]	A large urban teaching hospital located in the west side of Chicago, serving a large proportion of ethnically diverse and traditionally underserved patient population, USA	To conduct a preliminary evaluation of the Bridge-SU intervention on health services utilization and cost and to explore whether the intervention effect size differed by patient characteristics	Patients aged 18 and over, with five or more hospital admissions	586
*SDoH domains mentioned: in most studies this information was presented as a partial list given several examples or an enumeration ending in etc. Therefore, the information presented here is likely incomplete.				

Note: Except * Canada and ** UK, all other study countries are USA

The reviewed studies investigated various interventions to enhance healthcare outcomes and manage costs. Among these, home delivery of tailored meals to address food insecurity was evaluated for its potential to reduce healthcare utilization and expenditures [16]. Social worker-led care coordination intervention was examined for its effect on 30-day readmission rates [18]. Additionally, community health worker programs were assessed for their impact on outcomes for individuals with chronic conditions, with several studies focusing on these programs’ effectiveness [26, 27, 28, 44]Community-based models were also a significant focus. For example, the I-CAN Model aimed to reduce preventable healthcare utilization among underserved populations by addressing social determinants of health and fostering community partnerships [40]. Similarly, the Bridges to Care (B2C) program in a large academic medical centre in an urban low-income community sought to improve outcomes related to emergency department use, hospital admissions, and primary care access through an ED-initiated, multidisciplinary approach [49].

Interventions were implemented in various settings, including urban hospitals, academic medical centers, community-based programs, and safety-net clinics. Data collection and use related to social needs occurred in diverse healthcare environments such as emergency departments, primary care clinics, and inpatient hospital settings. The interventions targeted specific populations, including frequent users of emergency departments and inpatient care, uninsured individuals, and those with particular social needs or risks (e.g., homelessness, immigrant status, low socioeconomic status). This often involved targeting adults with multiple chronic conditions, low-income individuals, Medicaid and Medicare beneficiaries, as well as groups like veterans and the homeless. The outcomes evaluated included various metrics of healthcare utilization, such as emergency department visits and hospital admissions, alongside associated costs and patient satisfaction. None of the studies reviewed implemented social needs data collection and use at the population level or across entire health systems.

Table 3 below provides a summary of the key findings from the included studies regarding approaches and interventions for addressing social needs, along with their impacts on health services utilization and costs. A full summary can be found in Supplementary File B.

**Table 3:** Summary of approaches and interventions for addressing social needs: impacts on health services utilization and costs

Author (year)	SDoH data collection approach	SDoH domains mentioned*	Intervention	Summary	Impact on health services utilization?	Health services utilization outcomes	Impact on healthcare costs?	Healthcare costs outcomes
Berkowitz et al. (2018) [16]	Physician assessed. No approach tool mentioned	Food security (nutritional risk)	Medically-Tailored Meals and Meals on Wheels	Provides home deliveries of either medically-customized meals or non-tailored food to meet dietary needs	Yes, fewer ED visits and inpatient admissions	MTM program participants had fewer emergency department visits (Incidence Rate Ratio [IRR] 0.30; 95%CI 0.20 to 0.45) than matched non-participants, as did NTF program participants (IRR 0.56; 95%CI 0.47 to 0.68). MTM program participants also had fewer inpatient admissions (IRR 0.48; 95%CI 0.26 to 0.90).	Yes, lower medical expenditure	MTM program participants had lower medical expenditure (difference -$572, 95% CI -$933 to -$210). NTF program participation was associated with lower medical expenditure (difference -$159, 95%CI -$310 to -$8).
Bradley et al. (2018) [17]	Student teams assessed on intake, on each visit, after 12 visits and at discharge in weekly chart notes, with multiple choice questions	Housing, health insurance, transportation, employment, safety/security, income and stability of determinants	Interprofessional Care Access Network (I-CAN)	Student teams assist clients with healthcare navigation, appointment scheduling, transportation, and provider communication	Yes, decrease in ED visits, emergency medical services and hospitalization, improved access to primary care	For 38 participants, substantial reductions compared to the 6 months before I-CAN in the aggregate number of emergency department visits (37 vs. 10), emergency medical service calls (25 vs. 8), and hospitalization (12 vs. 3). Self-reported access to primary care improved (49% to 63%)	Yes, lower costs for ED visits, hospitalization, and emergency service calls	Estimated cost savings for the 38 clients, based on minimal estimated costs for number of ED visits, emergency medical service calls and hospitalization alone, were over $224,000 ($5,894 each)
Bronstein et al. (2015) [16]	Master of Social Work interns performed individualized needs assessments during post-discharge follow-ups. No tool mentioned	Medication access, transportation, home care needs, home safety, and behavioral barriers to care	Post-Discharge Care Coordination	Master of Social Work interns make follow-up calls and home visits to support patients after discharge, ensuring timely resource access	Yes, reduced likelihood of readmission to hospital	Improve likelihood of not being readmitted by 22% (RR = 1.222; 95% CI = 1.063–1.405). Highly significant risk improvement (χ2 = 8.99; *p* = 0.003)	Not reported	Not reported
Capp et al. (2017) [19]	Community advocacy liaison conducted a semi-structured interview during ED/discharge to assess patient’s healthcare experiences and needs	Housing, insurance/benefits, transportation, refugee services, care coordination, medication access	Bridges to Care (B2C)	Offers intensive medical, behavioral, and social care coordination with home visits and a multidisciplinary team post-ED visit or discharge	Yes, decrease in ED visits and hospitalizations and increase in primary care visits	Six months post intervention, significantly fewer ED visits (mean difference: 0.821, *p* < 0.05; a reduction of 27.9%) and hospitalizations (mean difference: 0.270, *p* < 0.1; a reduction of 15.5%) and significantly more primary care visits (mean difference: 1.307, *p* < 0.05; an increase of 114.0%), compared to patients in the control group	Not reported	Not reported
DeLaVega et al. (2022) [20]	Clinic staff used an 8-domain social needs survey every six months	Housing, food security, medication access, transportation, utilities, employment, education, caregiving	Resource Connection and Pharmacy Care	Provides patients with resource information and enhanced pharmacy care focusing on medication and social needs	Yes, decrease in ED visits and inpatient hospital admissions for both groups	Both groups experienced a decrease in ED visits and hospital admissions after 12-months, 129 (70.9%) vs 118 (64.8%) for screening and resources list, and 136 (74.7%) vs 110 (60.4%) for enhanced pharmacy. No significant difference between the two groups	Not reported	Not reported
Finkelstein et al. (2020) [21]	Screening for medical and social complexity noted in electronic medical record and written notes. No tool mentioned	Homelessness, social support, barriers to care	Camden Core Model	A comprehensive post-discharge program offering home visits, care coordination, and navigation of medical and social services	No, no significant difference between intervention and control group in 180-day hospital readmissions rates	The 180-day readmission rate was 62.3% in the intervention group and 61.7% in the control group. The adjusted between-group difference was not significant (0.82% points; 95% confidence interval, 5.97 to 7.61)	Not reported	Not reported
Gupta et al. (2023) [22]	Social determinants of health 13-item screener with validated question entered into the EMR	Food security, housing, utilities, transportation, financial stability, violence/abuse, education, language, health literacy, social support	EMR-Triggered Referrals	Automatically triggers digital prescriptions for social needs referrals based on electronic medical record data	Yes, decrease in primary care visits	Patients receiving increasing volume of referrals was significantly associated with fewer primary care visits (−0.071, *p* = 0.002), but comorbidities moderated this effect	Not reported	Not reported
Haggerty et al. (2023) [23]	Volunteer navigators used postal code (area deprivation index) plus brief discussions	Transportation, area-level material and social deprivation, barriers to care	Volunteer Navigator Outreach	Provides new patients with essential information and preparation tips for their first visit through a welcome call and email	Yes, positive first visit with new primary care provider, increase in number of primary care visits	Post intervention increase in patients’ abilities to seek, reach and engage with care and helped them attach successfully to newly-assigned family doctors. Increase in primary care visits (2.00 at 3 months post intervention, *p* = 0.04 vs. 1.2 in last 6 months at baseline). Decrease in ED use not significant	Not applicable	
Heisler et al. (2022) [24]	A community health worker conducted a comprehensive social needs assessment summarized data in a brief encounter form. No tool mentioned	No domains mentioned.	Community Health Worker Program	Conducts health, behavioral, and social needs assessments, creates action plans, and links participants to necessary services	Yes, lower ED visits	Lower adjusted ratios of ED visits (adjusted rate ratio = 0.96; *p* < 0.01) over 12-months, compared to the usual-care group	Mixed, lower ED visit costs, higher ambulatory care costs, no difference in inpatient or total costs	Lower adjusted ratios of ED visit costs (adjusted rate ratio = 0.96; *p* < 0.01).), but higher adjusted ratios of ambulatory care costs (adjusted ratio ratio = 1.15; *p* < 0.01). No differences in inpatient or total costs compared with the usual-care group
Henschen et al. (2022) [25]	Interdisciplinary team conducted an in-depth psychosocial assessment	Housing, food security, access to medication, transportation	Complex High Admission Management Program (CHAMP)	An interdisciplinary team addresses medical and social needs through comprehensive care planning and community visits	No, increase in inpatient 30-day readmissions, no difference in hospital admissions, total hospital days, ED visits and outpatient clinic visits	After 180 days, CHAMP patients had more inpatient 30-day readmissions [CHAMP incidence rate 1.3 (95% CI 0.91.8) vs. control 0.8 (95% CI 0.51.1), *p* = 0.04], though both groups had fewer readmissions compared to 180 days prior to enrollment. No differences in hospital admissions, total hospital days ED visits and outpatient clinic visits	Not reported	Not reported
Kangovi et al. (2017) [26]	Community health workers used a semi structured interview guide	Food security, housing, drug and alcohol use, social support	IMPaCT Intervention (Tailored Support)	Community health workers offer six months of tailored support, goal-setting, and long-term resource connections with coordination with primary care.	Yes, lower hospitalizations (but not significant)	At 6-months, 16% of intervention group were hospitalized vs. 17.8% in the usual care group (*p* = 0.68). By 1 year, 23.3% of patients in the CHW arm were hospitalized versus 31.6%in the goal-setting arm (*p* = 0.11). At 1 year, there were 68 total hospitalizations (278 hospital days) in the CHW arm versus 98 (414 hospital days) in the goal-setting arm (*p* = 0.17).	Not reported	Not reported
Kangovi et al. (2018) [27]	Community health worker conducted semi structured interview (open-ended) to assess socioeconomic determinants of health	Food security, housing, drug and alcohol use, family stress, trauma	IMPaCT Intervention (Social Support)	Delivers goal-setting and six months of tailored support, including social services and long-term support connections.	Yes, lower mean number of repeat hospitalizations and 30-day readmission (significant); fewer total days spent in hospital, shorter length of stay, fewer hospitalizations (not significant)	At 6 months, fewer total days spent in hospital for intervention group (155 days vs 345 days; absolute event rate reduction, 69%) and 9 months (300 days vs 471 days; absolute event rate reduction, 65%). Shorter average length of stay (difference, 3.1 days; 95% CI, 6.33 to 0.22; *p* = 0.06) and a lower mean number of hospitalizations (difference, 0.3; 95% CI, 0.6 to 0.0; *p* = 0.07) among hospitalized patients. Lower odds of repeat hospitalizations (OR, 0.4; 95% CI, 0.2–0.9; RD, 0.24; *p* = 0.02), including 30-day readmissions (OR, 0.3; 95% CI, 0.1–0.9; RD, 0.17; *p* = 0.04).	Not reported	Not reported
Kangovi et al. (2020) [28]	Community health workers used a semi structured interview guide to understand social and behavioral determinants of health	Food security, housing, drug and alcohol use, social support	IMPaCT Intervention (Tailored Coaching)	Provides six months of tailored coaching, social support, and long-term connections with resources through community health workers.	Not reported	Not reported	Yes, every dollar invested in the intervention would return $2.47 within a year	Total savings for Medicaid $1,401,307.99. This savings divided by program expenses ($567,950.82) yielded a return of $2.47 for every dollar invested, realized within a single fiscal year.
Kenyon et al. (2016) [29]	As part of midwife routine care, collection of demographic data and systematically assessed social risk factors and area deprivation quintile based on postal code	Housing, teen parenting, smoking, language (English), benefits, immigration status, social support, mental illness, area deprivation, ethnicity, access to social services, drug and alcohol use, violence/abuse	Pregnancy Outreach Worker Service	Offers case management and support from enrollment to six weeks postpartum, including home visits and resource management.	No, no difference antenatal attendance, routine child assessment or primary immunization uptake.	Antenatal attendance: No difference between groups, either for all women or for women with two or more social risk factors (10.1 vs 10.1 (mean difference; MD) 0.00, 95% CI (95% CI 0.37 to 0.37)). Routine child assessment attendance and primary immunization uptake: not difference between groups	Not reported	Not reported
Kitzman et al. (2022) [30]	Data based on zip code and screening (no tool mentioned)	Area deprivation, gender, race, insurance	Baylor Scott & White Health and Wellness Center	One group receives primary care with wrap-around services, while the other receives additional wellness and social programs.	Yes, decrease in ED and inpatient visits	Overall, 14% reduction in ED visits (*p* = 0.003) and 43% fewer inpatient visits (*p* < 0.0001) in year after enrollment, but moderated by chronic disease risk factors.	Yes, lower overall ED costs and inpatient costs, with lower ED costs among intervention group	Overall, 23.2% reduction in ED costs (*p* = 0.0007) and 49.5% reduction in inpatient costs (*p* < 0.0001). Participants in primary care medical home and population health service group had 37% lower ED costs than participants in the primary care medical home only (*p* = 0.01).
Liss et al. (2019) [31]	A study programmer queried health system databases to collect data on patients sociodemographic characteristics and a post- discharge survey	Psychosocial needs, no specific domains mentioned	Transitional Care	Includes post-discharge appointments with a multidisciplinary team to assess and address patients’ medical and psychosocial needs	Yes, lower inpatient admissions at 90 and 180 days	Transitional care patients had 37 and 35% lower probability of any inpatient admission over 90 days (RR0.63; 95% CI 0.430.91) and 180 days (RR 0.65; 95% CI 0.470.89) and 42% fewer inpatient admissions over 180 days (incidence rate ratio 0.58; 95% CI 0.370.90). No significant difference between arms in the 90-day probability of death or additional hospital encounters (relative risk [RR] 0.89; 0.91; 95% confidence interval [CI] 0.741.13)	Not reported	Not reported
Losonczy et al. (2017) [32]	Undergraduate volunteers screened patients and asked them to prioritize their top 3	Social, economic, environmental, and legal needs: violence, housing, education, transportation, income, immigration, refugee, language, access to care, caregiving	Highland Health Advocates	An ED-based help desk and medical-legal partnership where volunteers assist with public resources, legal referrals, and medical home connections	Mixed, increase in having a medical home and doctor, no difference in ED utilization	At 1 month, increase in linkage with medical home in intervention group (92% vs 76%). At 6 months, more subjects in the intervention group had a doctor (93% v 69%). No difference was found in ED utilization	Not reported	Not reported
Moreno et al. (2021) [33]	Home-based care provider conducted a standardized comprehensive assessment to identify social needs, strengths and barriers	Activities of daily living, insurance, access to resources, caregiver support, visual or hearing needs, culturally restricted treatments, access to care, transportation, income, psychosocial factors	Home-Based Social Intervention	Delivers initial assessments, care coordination, and social service connections over six months through a social worker and community health worker	Yes, decrease in acute hospitalization and ED visits	After 12 months, pre/post reductions acute hospitalizations (Mean −0.66, IRR = 0.82, *p* = 0.006) and ED visits (Mean −0.57, IRR = 0.67, *p* = 0.003), compared to matched comparators	Not reported	Not reported
Onwuanyi et al. (2020) [34]	Population health tool (Healthy Planet) used in the electronic medical record system to collect comprehensive social and behavioral data	Financial barriers to medication	Social Determinants of Health Interventions	Provides interventions targeting specific social determinants alongside usual medical care without detailed descriptions.	Yes, reduction in 30-day readmissions and shorter length of stay	Less likely to be readmitted in 30 days, had shorter stay in the hospital and lower mortality (no statistics reported)	Not reported	Not reported
OToole et al. (2016) [35]	Comprehensive assessment of needs. No tool or approach mentioned	Homelessness, legal needs, access to care, food security, hygiene, clothing, employment, education, disability, transportation, social support	Homeless Patient Aligned Care Team (H-PACT)	Offers integrated care with open-access, outreach services, and sustenance support for homeless individuals	Yes, reduced ED visits and hospitalizations	After 6 months, 19.0% reduction in ED use and a 34.7% reduction in hospitalizations	Not reported	Not reported
Pantell et al. (2020) [36]	Navigators administered baseline household social risk questionnaire	Food security, utilities, employment, environment, ability to pay medical bills, insurance, income, access to resources, access to care, benefits	Navigator Intervention	Assists caregivers with resolving social needs through bi-weekly contact and access to targeted resources for up to three months	Mixed, decrease in risk of hospitalization, no difference in ED visits	After 1 year, there was no difference in risk of an emergency department visit between the 2 groups. Children enrolled in the in-person navigator group had a decreased risk of hospitalization within 12 months (hazard ratio, 0.59; 05% CI, 0.38–0.94; *p* = 0.03), making them 69% less likely to be hospitalized	Not reported	Not reported
Pruitt et al. (2018) [37]	Self-reported social needs	Transportation, utilities, food security, visual needs, benefits, access to medication, housing, access to resources	WellCare’s Health Connections	A call center-based program providing social service referrals to address needs	Not reported	Not reported	Yes, decrease overall expenditure	In the second year, decrease in mean expenditures for the group of participants who reported all their social needs met was $2443 (10%) greater than the group who reported none of their social needs met.
Roth et al. (2023) [38]	Validated questions embedded into existing clinical workflows to systematic screening by primary care team	Food security, utilities, transportation,	Providence Diabetes Collective Impact Initiative (DCII)	Includes outreach, diabetes education, social needs screening, and referral to an on-site community resource desk	Mixed, increase in virtual primary care visits, no difference in primary care, ED, or inpatient utilization	Increase in the average number of virtual primary care visits of 0.35 per member, per year (*p* < 0.001) compared to controls. No significant differences in the likelihood of an inpatient or emergency department, primary care, inpatient, and emergency department utilization	Not reported	Not reported
Rucker et al. (2023) [39]	BearSCREEN (Survey of Comprehensive Risk Evaluation and Emerging Needs), a 13- item computerized survey completed via tablet using REDCap software	Literacy, housing, education, food security, risk for human trafficking, sexual health, safety, access to care, substance abuse, immigration, violence/abuse, legal needs, mental health	General and Tailored Resource Referral	Provides a general list of community resources, with tailored navigation and plan development for the intervention group	No, no difference in ED visits	After 12 months, no difference in number of subsequent ED visits for any visit type (46.5% vs. 46.2%; adjusted odds ratio [aOR] 1.0 [95% confidence interval {CI}: 0.7, 1.5]), or an SSV (17.7% vs. 21.5%; aOR 0.7 [95% CI: 0.4, 1.2])	Not reported	Not reported
Schickedanz et al. (2019) [40]	Phone screening: 14-question social needs questionnaire, follow by full assessment (10–15 minutes) if at least 1 social need identified	Health literacy, education, financial security, food security, housing, employment, access to resources, transportation, caregiver support, social support	Health Leads	Offers telephonic social needs screening, navigation, and referral with immediate or follow-up provision of resource information	Yes, decrease in ED, outpatient, and inpatient visits	After 1 year, 2.2% decrease in total visit count for intervention group (outpatient, ED and inpatient) (95% CI 4.5%, 0.1%; *p* = 0.058). ED visit count decreased by 0.06, inpatient hospitalizations by 0.08 and ambulatory visits by 0.03 for intervention patients compared with controls. Effect was larger in all low socioeconomic group receiving the intervention (7.0 to 12.1% decrease)	Not reported	Not reported
Schumacher et al. (2017) [41]	Health literacy screening (Rapid Estimate of Adult Literacy in Medicine)	Health literacy	ED-to-Home Intervention	Coaches assist with follow-up visits, disease recognition, medication reconciliation, and provider communication post-discharge.	No, no difference in primary care doctor visits	No difference in post ED follow-up doctor visits	Not reported	Not reported
Sege et al. (2015) [42]	Questions to determined upstream factors. No tool or approach mentioned	Food security, housing, utilities, hardship, legal needs	Developmental Understanding and Legal Collaboration for Everyone (DULCE)	Provides support and resource access through home visits and communication with the family and medical provider until six months post-enrollment	Yes, increase in routine preventive care, decreased ED visits	By age 1, intervention infants were more likely to have 5 or more routine preventive care visits (78% vs 67%, *p* = 0.01), 93% (vs. 86%) of intervention families continued to receive primary care at the study site (*p* = 0.056). Likelihood of ED visits was significantly lower in intervention group at 6 months (36.5% vs 49.7%, *p* = 0.021), but not significant at 12 months (59.3% vs 65.0%, *p* = 0.40). Total ED visits was lower at 6 months (*p* = 0.023) but not at months (*p* = 0.08)	Not reported	Not reported
Smith et al. (2021) [43]	Electronic medical record screening	Homelessness, frequent ED use, chronic pain	Frequent User System Engagement (FUSE)	Coordinates care and stable housing for frequent hospital users with a focus on multidisciplinary support and community resource referrals	Yes, decrease in ED visits, increase in primary care provider visits and diagnostic tests	After 18 months, significant decrease in ED visits (χ2 (5, *n* = 11) = 13.47, *p* = 0.02) a, increase in primary care provider visits (χ2 (5, *n* = 11) = 30.12, *p* < 0.01), and increase in number of diagnostic tests (χ2 (5, *n* = 11) = 27.37, *p* < 0.01)	Mixed, no difference for ED and total costs, increase for diagnostic tests	Significant increase in costs of diagnostic tests (χ2 (5, *n* = 11) = 16.08, *p* < 0.01). Differences in ED costs (χ2 (5, *n* = 11) = 10.44, *p* = 0.06) and total costs (χ2 (5, *n* = 11) = 8.34, *p* = 0.14) were not significant
Vasan et al. (2020) [44]	Community health workers used a semi structured interview guide to understand social and behavioral determinants of health	Food security, housing, drug and alcohol use, social support	IMPaCT (General Support)	Community health workers provide goal setting, social support, and health behavior coaching with connections to long-term resources	Yes, decrease in hospitalizations and length of stay	Over 9398 observed patient months (1 to 12 months), total number of hospital days per patient in the intervention group was 34% lower (IRR) 0.66, *p* < 0.0001)). This reduction was driven by fewer hospitalizations per patient (0.27 vs 0.34, *p* < 0.0001) and shorter mean length of stay (4.72 vs 5.57 days, *p* = 0.03). Rates of hospitalization also decreased outside patients’ primary health system (18.8% vs 34.8%, *p* = 0.0023)	Not reported	Not reported
Vest et al. (2018) [45]	Based on provider assessment of social needs or proactive review of records of patients with scheduled appointments	No domains mentioned	Eskenazi Health Wraparound Services	Offers integrated services such as social work and behavioral health at outpatient clinics to address social determinants of health	Yes, decrease in ED visits and hospitalization	After 1 year, 7% decrease in expected number of hospitalizations and 5% decrease in ED visits. Number of nonemergent ED visits was lower, but not significant	Yes, decrease in costs from hospitalizations	Estimated cost savings from potentially avoided hospitalizations alone was $1.4 to $ 2.4 million annually ($76–$131 per person)
Wallace et al. (2020) [46]	ED registration staff collected data a 10-item social needs screening tool (adapted from Health Leads) in Spanish or English, adapted for 5th grade literacy and long-term risk over 12 months	Transportation, access to care, access to medication, ability to pay for basic needs, utilities, housing, employment, access to resources	Electronic Referral to 2–1–1	Provides direct electronic referrals to a comprehensive list of local providers for various social needs	No, no difference in hospitalization, increase in ED use	After 3 months, patients with at least 1 social need had a significant increase in ED use (1.07 before vs 1.36 after, *p* = 0.03) while patients with no needs had an increase in primary care visits (0.24 before vs 0.56 after, *p* = 0.03). ED visits increased among those who received follow-up and referrals from 2 to 1–1 (1.97 before vs 2.56 after, *p* = 0.006). No differences in hospitalizations	Not reported	Not reported
Weerahandi et al. (2015) [47]	Comprehensive psychosocial assessment during the index hospitalization	No domains mentioned	Preventable Admissions Care Team (PACT)	A social work model offering tailored interventions, including phone calls, home visits, and appointment accompaniment post-discharge.	Yes, decrease in hospital readmissions	Compared to controls, 30-day readmission rate decreased by 34% (*p* = < 0.001), 60-day hospitalization rate decreased by 22% (*p* = 0.004); 90-day hospitalization rate decreased by 19% (*p* = 0.006).	Yes, lower overall costs	Inpatient costs 30 days post-index were $2.7 million for PACT patients and $3.6 million for controls.
Woodhead et al. (2017) [48]	Identified by practice staff or self-identified by patients	Financial needs, benefits, education, employment, housing	Primary Care Co-located Debt and Welfare Advice	Provides specialist advice on welfare benefits and debts within general practices	No, no changes in primary care visits	No changes in 3-months consultation rates with general practitioner	Yes, financial benefit to recipient returns on investment	Per capita, advice recipients received £15 per £1 of funder investment.
Wu et al. (2019) [49]	Screening for high risk through a predictive model or risk identified by their provider	No domain mentioned	Baltimore Community-Based Organizations Neighborhood Network (CONNECT)	Enhances capacity with an online tool for community resource referrals and meet-and-greets between organization staff and healthcare staff	No, no difference in ED visits or hospital days	There was no significant effect of the intervention on healthcare utilization outcomes, including ED visits and days spent in hospital	Not reported	Not reported
Xiang et al. (2019) [50]	Biopsychosocial needs assessment. No approach or tool mentioned	Health literacy, social support, access to care, housing, transportation	Bridge Model for Super Utilizers (Bridge-SU)	A transitional care model for frequent hospital users addressing medical and social needs through coordinated care and case management	Yes, decrease in hospital admissions, readmissions, and ED visits	After 12 months, significant reduction in the total number of hospital admissions (pre 5.76 (95% CI = 5.64–5.88) vs post 2.38 (95% CI = 2.17–2.59), nearly halved 30-day readmission rates (pre: 25.5% (95% CI = 24.7–26.4%) vs post 13.4% (95% CI = 12.0–14.8%), and number of emergency department visits (pre: average of 5.39 visits (95% CI = 5.09–5.68) vs. post 3.38 visits (95% CI = 2.78–3.98)	Yes, decrease in hospitalization costs	After 12 months, significant reductions in average hospital charges per episode by $14,150, and total hospital charges per person decreased by nearly $200,000

SDoH domains mentioned: in most studies this information was presented as a partial list given several examples or an enumeration ending in etc. Therefore, the information presented here is likely incomplete

### Screening and interventions

Approaches to screening for social needs were inconsistently reported and often not described in detail. Some used standard surveys [19, 20, 23, 30–37, 39, 40, 50] administered to patients, while others used information extracted or derived from electronic medical records or other databases [21, 31, 42]. Other studies implemented screening for social needs via an assessment by a member of the healthcare team [16, 38, 44, 46], such as a community health worker, a student, or a similar role, trained to explore social needs with patients [18–21, 24–27, 36, 43, 49, 49, 50] A few studies had used a patient’s address to assign area-level data (e.g. using a postal code to assign a patient to a certain level of deprivation) [17, 22, 29].

Studies often screened for multiple needs simultaneously, including: food security [16, 19, 20, 23, 25, 30, 34, 34, 35, 36, 37, 39, 41, 43], housing [19, 21, 23, 25, 29, 30, 34, 35, 36, 37, 39–43, 46, 48–50], employment [30, 34, 36, 40, 46, 48], income [40, 48, 50], transportation [18–20, 25, 30, 32, 34–37, 40, 46, 49], home safety [18, 47, 49], health insurance [22, 32, 36, 40], social/disability benefits [22, 29, 35, 36, 49], immigrant or refugee status [29, 39, 49, 50], ability to pay for utilities [19, 20, 35–37, 41, 46], ability to pay for medication [25, 33], caregiver responsibilities/needs [20, 30, 32–37, 39, 45, 46, 50], education [19, 20, 30, 39, 48, 50], language [29, 50], health literacy [19, 23, 39, 47], social support [19, 21, 23, 26, 27, 29, 30, 34, 43, 47], violence/abuse/trauma [26, 27, 39, 43, 50], gender [22], race [22], and disability status [30, 32, 36]. Most studies only provided examples or incomplete enumerations of domains for which data were collected.

Information about patients’ needs was used to help address social needs through interventions of various intensity, and sometimes combining multiple components including referring patients to relevant community or social resources (e.g., navigator, printed resources lists) [17, 19, 20, 36, 37, 39, 44, 46, 47, 50], co-located services (e.g., medical-legal partnership, community help desk) [22, 37, 38, 41, 48, 50], outreach services [30], providing direct resources to address needs (e.g., food delivery or vouchers, housing) [16, 22, 42], community health worker programs [23, 26, 27, 32, 41, 43], care coordination during transitions in care [18, 21, 31, 32, 40, 49] and case management programs for specific clienteles [25, 29, 42].

### Impact on health service utilization

Related to ED, 13 studies reported a decrease in the number or likelihood of ED visits [16, 20, 22, 24, 30, 32, 34, 38, 40–42, 47, 49], while six found no difference [25, 34, 36, 37, 44, 50]. Only one study reported an increase in ED use [46]. One study reported decreased use of emergency medical calls [40]. With regards to primary care, three reported improved access to primary care [17, 40, 50], four found an increase in primary care visits following the intervention [17, 20, 42, 49], one reported an increase in preventive care [41], one found an increase in virtual visits [37], and another reported an increase in diagnostic tests [42]. In contrast, four found no changes in primary care attendance [23, 29, 37, 48]^,^ and one reported a decrease in primary care visits [19]. For outpatient care, two studies reported no difference in visits [25, 37], while another reported a decrease in visits [34].

For hospitalizations, 14 studies found a decrease in hospitalizations [16, 18–20, 20–22, 24–27, 30, 32, 34, 36, 38, 40, 43, 47, 49]. The effect of interventions on readmission rates shows a mixed pattern. Five reported reduced readmissions [18, 27, 31, 45, 47]. Three reported shorter length of stay [27, 33, 43]. In contrast, four studies reported no difference in hospitalizations [25, 37, 39, 46], while two found no difference in readmission rates compared to control groups [21, 25], and two others found no change in the number of hospital days [44, 47].

### Impact on healthcare costs

Most studies reported reductions in healthcare costs associated with the interventions. Specifically, four studies documented lower total medical costs [16, 28, 45, 48] three reported decreased costs for ED visits [22, 24, 40] and six found reductions in hospitalization costs [22, 24, 31, 38, 40, 47]. Additionally, two studies noted positive returns on investment [28, 48]. In contrast, one study reported no difference in ED costs [42], and another found higher diagnostic test costs [28]. Another study found no changes in inpatient or total costs but observed higher ambulatory care costs [24].

### Study quality assessment

Of the 35 included studies, 4 studies (11%) were rated as high quality, 17 studies (49%) were rated as medium quality, and 14 studies (40%) were rated as low quality. Many studies were classified as low quality due to limited reporting of methods, with numerous “Can’t Tell” appraisals similar to “No” responses for quality items. Due to resource constraints, it was not possible to contact study authors to clarify the “Can’t Tell” appraisals. Supplementary file C provides the full quality assessment.

## Discussion

This systematic review evaluated the impact of collecting and using social needs data on health service utilization and associated costs. The findings suggest that integrating social needs data into healthcare settings can influence health service utilization and healthcare costs, with varying degrees of effectiveness depending on the context and models of intervention. This finding is consistent with a previous literature review [11].

### ED utilization

The majority of reviewed studies (13 studies) [16, 20, 22, 24, 30, 32, 34, 38, 40–42, 47, 49] observed lower ED utilization associated with social needs-informed interventions. These studies often involved higher-intensity models (e.g., community health workers, enhanced interprofessional care teams, proactive care coordination). In contrast, six studies [25, 34, 36, 37, 44, 50] reported no change in ED utilization or mixed results with interventions, most describing lower intensity approaches such as resource handouts or simple referrals. One study [46] that relied on electronic referrals to community-based resources reported increased ED visits; qualitative feedback from clinicians suggested the intervention was preferentially offered to patients perceived to have greater social needs, which could contribute to higher subsequent ED use (selection bias). Overall, these findings suggest screening for social needs in the ED may be associated with lower ED utilization, but screening without proactive support to address these needs may be insufficient and may face implementation challenges. In a review of interventions targeting the elderly to reduce ED utilization, Fan et al. (2014) [51] also noted mixed intervention effectiveness and found that critical elements, including multidisciplinary teams, integrated primary care, and social care, often existed in effective interventions in contrast to significantly ineffective ones. Given the heterogeneity in interventions, study designs, populations and reporting, findings should be interpreted with caution. [52]

### Primary care

Although findings for primary care were mixed, most studies reported improved access, more primary care visits or increased preventative care [17, 20, 37, 40, 41, 42, 49, 50]. In contrast, other studies found no change [23, 29, 37, 48] or decrease in visits [19]. Many of the studies reporting improve access or an increase in primary care utilization targeted patients with unmet needs or access barriers. Overall, these findings suggest that social needs-informed interventions may help shift care toward less-resource intensive primary care or address unmet care needs when dedicated support is present (e.g., navigation or community health workers) for patients experiencing barriers to primary care access, but variability in interventions and underreporting of screening limit the conclusions that can be drawn. Others have suggested that implementing systematic screening and linkage to community resources can enhance awareness of social needs and facilitate greater access to services [53]. The only three studies from outside the USA (Canada and the United Kingdom) were primary care-based [17, 29, 48], which may possibly reflect that universal healthcare systems may be structurally predisposed to integrate social needs screening in primary care. There were very few studies from outside the USA which suggest slow uptake of such approaches internationally. Future studies are warranted to evaluate social needs-informed interventions in different contexts and to determine whether the most sustained impacts can be obtained in primary care.

### Hospital utilization

Most studies reporting on hospital utilization found a decrease in admissions, readmissions or length of stay [16, 18–20, 21–22, 24–27, 30, 32, 34, 36, 38, 40, 43, 47, 49], although seven reported no change or mixed results [21, 25, 37, 39, 44, 46, 47]. Studies reporting decreased hospital utilization generally targeted patients in high-poverty areas, who were uninsured or had more complex health needs (e.g. older adults, chronic disease) with models such as community health workers or social worker-supported care transitions, enhanced interprofessional care or direct provision of resources (e.g., meals). Interventions that did not produce significant changes were often more targeted to frequent users and often featured care coordination or post-discharge follow-ups. Contextual factors and implementation strategies may influence this variability in the effectiveness of interventions [51]. Despite the variability, the notable success in reducing hospitalizations through implemented interventions highlights the importance of tailoring and customizing approaches to specific contexts and populations.

### Healthcare costs

Our review found that most studies highlight cost reductions [16, 22, 22, 24, 24, 28, 31, 38, 40, 45, 47, 48], although three studies indicated no significant cost changes or even increases in areas like diagnostic testing and ambulatory care [24, 28, 42]. The impact of collecting and using social needs data may vary depending on the specific type of intervention and the characteristics of the patient population involved [54]. Some have suggested that ED visits by populations with high levels of social needs might be more effectively managed through primary and community healthcare services, thereby saving cost [55].

### Interpretation

The results suggest that collecting social needs data and addressing them through related interventions may positively impact healthcare utilization and costs. Studies reporting positive outcomes may have successfully identified social needs and helped address these needs through referrals to community resources or case management through early and tailored interventions, which are often drivers of increased healthcare utilization in the ED and hospitals.

Implementing social needs data screening in healthcare settings requires careful planning and resource allocation. None of the included studies had implemented population-wide screening. However, the variability in results underscores the importance of context-specific approaches, considering factors such as the healthcare setting, population demographics, and available resources. Standardized reporting and more robust study designs are needed to better understand these interventions’ long-term impact, best implementation strategies, and scalability.

### Limitations

Many studies’ limited reporting of methods contributed to lower quality ratings (49% of studies categorized as medium quality and 40% as low quality), indicating a need for more rigorous and transparent research practices in this area. Not contacting authors for clarification on “Can’t Tell” appraisals may have further impacted the assessment of study quality. The wide range of screening approaches, interventions, settings, study design, quality and reporting may affect the certainty of these findings and preclude any estimation of the effect size of the impact on utilization and costs. Because the approaches to collecting social needs data varied widely, were rarely detailed and inconsistently reported, it was impossible to adequately assess which approaches were most effective in reducing utilization and costs, and this may limit the generalizability of our findings.

Most studies (32/35) were conducted in the USA, which may limit their generalizability to other contexts. The literature in the area is heterogeneous, with inconsistent use of keywords and challenging to search given its emerging nature. Although we rigorously followed PRISMA guidelines and supplemented our search strategy with AI-assisted snowballing strategies, we may have missed relevant studies. It is also possible that community-based settings are underrepresented in our review, as they may not formally collect social needs data.

## Conclusion

This review highlights the potential benefits of systematically collecting and using social needs data in healthcare settings to reduce healthcare utilization and associated costs. However, the variability in study quality, design, and reporting underscores the need for more robust evidence. While social needs interventions appear promising, especially for targeted populations with high levels of social needs, there is a lack of high-quality evidence on their broad implementation across general populations or entire health systems. Future initiatives should focus on piloting social needs data collection and interventions at scale, with careful evaluation to ensure they achieve the intended outcomes without unintended consequences.

## Electronic supplementary material

Below is the link to the electronic supplementary material.

Supplementary material 1

## Data Availability

No datasets were generated or analysed during the current study.

## Abbreviations

ED: Emergency Department
EHR: Electronic Health Record
EMR: Electronic Medical Record
MMAT: Mixed methods Appraisal Tool
PRISMA: Preferred Reporting Items for Systematic reviews and Meta-Analyses
