# Supplementary material for: Collecting and using social needs data in health settings: a systematic review of the literature on health service utilization and costs
Source: BMC Health Serv Res. 2025 Sep 30;25:1258. doi: 10.1186/s12913-025-13458-2 (PMC12482085; doi:10.1186/s12913-025-13458-2)
Supplement: Supplementary file 1 — Supplementary material 1 [file 12913_2025_13458_MOESM1_ESM.docx]

**Supplementary file A: Detailed search strategies**

**Ovid MEDLINE: Epub Ahead of Print, In-Process & Other Non-Indexed Citations, Ovid MEDLINE® Daily and Ovid MEDLINE® <1946-Present>**
**Search Strategy:**
**1**  exp Health Services/ [ healthcare area terms ]
**2**  exp Ambulatory Care Facilities/
**3**  (community adj2 "health center$").mp.
**4**  (neighbo?rhood adj2 "health center$").mp.
**5**  (community adj2 "health centre$").mp.
**6**  (neighbo?rhood adj2 "health centre$").mp.
**7**  CHCs.mp.
**8**  CHC.mp.
**9**  (health$ adj2 clinic$).mp.
**10**  (health$ adj2 service$).mp.
**11**  exp Primary Health Care/
**12**  (primary adj1 healthcare).mp.
**13**  (primary adj1 "health care").mp.
**14**  (primary adj1 care).mp.
**15**  (patient-centered adj1 care).mp.
**16**  ("patient centered" adj1 care).mp.
**17**  (patient-focused adj1 care).mp.
**18**  ("patient focused" adj1 care).mp.
**19**  (patient-centred adj1 care).mp.
**20**  ("patient centred" adj1 care).mp.
**21**  exp Hospital/
**22**  (hospital$ adj3 care).mp.
**23**  (hospital$ adj3 service$).mp.
**24**  (inpatient$ adj3 care).mp.
**25**  (inpatient$ adj3 service$).mp.
**26**  (in-patient$ adj3 care).mp.
**27**  (in-patient$ adj3 service$).mp.
**28**  (outpatient$ adj3 care).mp.
**29**  (outpatient$ adj3 service$).mp.
**30**  (out-patient$ adj3 care).mp.
**31**  (out-patient$ adj3 service$).mp.
**32**  exp Ambulatory Care/
**33**  (ambulatory adj care).mp.
**34**  (urgent adj care).mp.
**35**  (clinic adj visit$).mp.

**36** safety-net.mp
**37** (safety adj net).mp
**38** "Federally Qualified Health Center".mp

**39**  FQHC.mp.
**40**  exp Emergency Service, Hospital/
**41**  (emergency adj department$).mp.
**42**  (emergency adj room$).mp.
**43**  (emergency adj ward$).mp.
**44**  or/1-43
**45**  Social Determinants of Health/ [ social determinants of health terms ]
**46**  (social adj determinant$).mp.
**47**  SDOH.mp.
**48**  SDH.mp.
**49**  (unmet adj1 need$).mp.
**50**  (under-met adj1 need$).mp.
**51**  (social adj1 need$).mp.
**52**  (economic adj1 need$).mp.
**53**  exp Educational Status/
**54**  (education$ adj2 status).mp.
**55**  (education adj2 achievement$).mp.
**56**  Employment/
**57**  Unemployment/
**58**  Poverty/
**59**  employ$.mp.
**60**  unemploy$.mp.
**61**  career$.mp.

**62**  occupation$.mp.
**63**  poverty.mp.
**64**  income.mp.
**65**  exp Food supply/
**66**  (food adj1 suppl$).mp.
**67**  (food adj1 assistance).mp.
**68**  (food adj1 securit$).mp.
**69**  (food adj1 insecurit$).mp.

**70**  exp Housing/
**71**  housing.mp.

**72**  homeless$.mp.

**73**  rent$.mp.
**74**  exp Public Assistance/
**75**  exp Social Welfare/
**76**  (public adj assistance).mp.

**77**  (social adj welfare).mp.
**78**  (social adj service$).mp.
**79**  Medicare.mp.
**80**  Medicaid.mp.
**81**  childcare.mp.
**82**  (child adj care$).mp.
**83**  (day adj care$).mp.

**84**  daycare$.mp.
**85**  (utility adj assistance).mp.
**86**  (medical adj legal).mp.
**87**  exp Insurance, Health/
**88**  (health adj insurance).mp.
**89**  exp Domestic Violence/
**90**  (domestic adj violence).mp.
**91**  (family adj violence).mp.
**92**  (child adj2 abuse).mp.
**93**  (elder adj abuse).mp.
**94**  (spous$ adj abuse).mp.
**95**  exp Transportation/
**96**  transport$.mp.
**97**  commute.mp.
**98**  commuting.mp.
**99**  exp Interpersonal Relations/
**100**  exp Social Isolation/
**101**  (interpersonal adj safety).mp.

**102**  (social adj2 interact$).mp.
**103**  (social adj2 relationship).mp.
**104**  (social adj2 isolat$).mp.
**105**  or/45-104
**106**  exp Electronic Health Records/ [ data terms ]
**107**  (electronic adj2 "medical record$").mp.
**108**  (electronic adj2 "health record$").mp.
**109**  (personal adj2 "medical record$").mp.

**110**  (personal adj2 "health record$").mp.
**111**  (medical adj2 "medical record$").mp.
**112**  (medical adj2 "health record$").mp.
**113**  EHR.mp.

**114**  EHRs.mp.
**115**  EMR.mp.

**116**  EMRs.mp.

**117**  PHR.mp.
**118**  PHRs.mp.
**119**  statistics & numerical data.fs.
**120**  or/106-119
**121**  Mass screening/ [ screening terms ]
**122**  screen$.mp.
**123**  (social adj history).mp.
**124**  "social risk? assessment?".mp.
**125**  "social need? identification".mp.
**126**  "social determinant? assessment".mp.
**127**  "social determinant? screening".mp.
**128**  "social determinant? evaluation".mp.
**129**  "social factor? assessment".mp.
**130**  "social factor? screening".mp.
**131**  "social determinant? identification".mp.
**132**  (socioeconomic adj screening).mp.
**133**  or/121-132
**134**  44 and 105 and 120 and 133
**135**  exp Animals/ not (exp Animals/ and Humans/) [ remove animal studies ]
**136**  134 not 135
**137**  limit 136 to (english language and yr="2015 -Current")
**138**  remove duplicates from 137

**Embase Classic+Embase <1947 to 2024 February 23>**
**Search Strategy:**
1 exp Health Service/ [ healthcare area terms ]
2 exp Outpatient Department/
3 (community adj2 "health center$").mp.
4 (neighbo?rhood adj2 "health center$").mp.
5 (community adj2 "health centre$").mp.
6 (neighbo?rhood adj2 "health centre$").mp.
7 CHCs.mp.
8 CHC.mp.

9 (health$ adj2 clinic$).mp.
10 (health$ adj2 service$).mp.
11 exp Primary Health Care/
12 (primary adj1 healthcare).mp.
13 (primary adj1 "health care").mp.

14 (primary adj1 care).mp.
15 (patient-centered adj1 care).mp.
16 ("patient centered" adj1 care).mp.
17 (patient-focused adj1 care).mp.
18 ("patient focused" adj1 care).mp.
19 (patient-centred adj1 care).mp.
20 ("patient centred" adj1 care).mp.
21 exp Hospital/
22 (hospital$ adj3 care).mp.
23 (hospital$ adj3 service$).mp.
24 (inpatient$ adj3 care).mp.
25 (inpatient$ adj3 service$).mp.
26 (in-patient$ adj3 care).mp.
27 (in-patient$ adj3 service$).mp.
28 (outpatient$ adj3 care).mp.
29 (outpatient$ adj3 service$).mp.
30 (out-patient$ adj3 care).mp.
31 (out-patient$ adj3 service$).mp.
32 exp Outpatient Care/
33 (ambulatory adj care).mp.
34 (urgent adj care).mp.
35 (clinic adj visit$).mp.
36 safety-net.mp.
37 (safety adj net).mp.

38 "Federally Qualified Health Center".mp

39 FQHC.mp.
40 exp Hospital Emergency Service/
41 (emergency adj department$).mp.
42 (emergency adj room$).mp.
43 (emergency adj ward$).mp.
44 or/1-43
45 "Social Determinants of Health"/ [ social determinants of health terms ]
46 (social adj determinant$).mp.
47 SDOH.mp.
48 SDH.mp.
49 (unmet adj1 need$).mp.
50 (under-met adj1 need$).mp.
51 (social adj1 need$).mp.
52 (economic adj1 need$).mp.
53 exp Educational Status/
54 (education$ adj2 status).mp.
55 (education adj2 achievement$).mp.
56 Employment/
57 Unemployment/
58 Poverty/
59 employ$.mp.
60 unemploy$.mp.

61 career$.mp.
62 occupation$.mp.
63 poverty.mp.
64 income.mp.
65 exp Food Insecurity/
66 (food adj1 suppl$).mp.
67 (food adj1 assistance).mp.
68 (food adj1 securit$).mp.
69 (food adj1 insecurit$).mp.
70 exp Housing/
71 housing.mp.

72 homeless$.mp.
73 rent$.mp.
74 exp Social Care/
75 exp Social Welfare/
76 (public adj assistance).mp.

77 (social adj welfare).mp.
78 (social adj service$).mp.

79 Medicare.mp.

80 Medicaid.mp.

81 childcare.mp.
82 (child adj care$).mp.
83 (day adj care$).mp.
84 daycare$.mp.
85 (utility adj assistance).mp.
86 (medical adj legal).mp.
87 exp Health Insurance/
88 (health adj insurance).mp.
89 exp Domestic Violence/
90 (domestic adj violence).mp.
91 (family adj violence).mp.
92 (child adj2 abuse).mp.
93 (elder adj abuse).mp.
94 (spous$ adj abuse).mp.
95 exp "Traffic and Transport"/
96 transport$.mp.
97 commute.mp.
98 commuting.mp.
99 exp Social Environment/
100 exp Social Isolation/
101 (interpersonal adj safety).mp.
102 (social adj2 interact$).mp.

103 (social adj2 relationship).mp.
104 (social adj2 isolat$).mp.
105 or/45-104
106 exp Electronic Health Record/ [ data terms ]
107 (electronic adj2 "medical record$").mp.
108 (electronic adj2 "health record$").mp.
109 (personal adj2 "medical record$").mp.
110 (personal adj2 "health record$").mp.
111 (medical adj2 "medical record$").mp.
112 (medical adj2 "health record$").mp.
113 EHR.mp.

114 EHRs.mp.
115 EMR.mp.
116 EMRs.mp.
117 PHR.mp.
118 PHRs.mp.

119 or/106-118
120 Mass screening/ [ screening terms ]

121 screen$.mp.

122 (social adj history).mp.

123 "social risk? assessment?".mp.
124 "social need? identification".mp.
125 "social determinant? assessment".mp.
126 "social determinant? screening".mp.
127 "social determinant? evaluation".mp.
128 "social factor? assessment".mp.
129 "social factor? screening".mp.
130 "social determinant? identification".mp.
131 (socioeconomic adj screening).mp.
132 or/120-131
133 44 and 105 and 119 and 132
134 Nonhuman/ [ remove animal studies ]
135 133 not 134
136 limit 135 to (english language and yr="2015 -Current")
137 remove duplicates from 136

**Cochrane CENTRAL**

#1 [mh "Health Services"]

#2 [mh "Ambulatory Care Facilities"]

#3 [mh "Primary Health Care"]

#4 [mh "Hospital"]

#5 [mh "Ambulatory Care"]

#6 [mh "Emergency Service, Hospital"]

#7 community NEAR/2 "health center" OR neighborhood NEAR/2 "health center" OR neighbourhood NEAR/2 "health center" OR community NEAR/2 "health centre" OR neighborhood NEAR/2 "health centre" OR neighbourhood NEAR/2 "health centre" OR community NEAR/2 "health centers" OR neighborhood
NEAR/2 "health centers" OR neighbourhood NEAR/2 "health centers" OR community NEAR/2 "health centres" OR neighborhood NEAR/2 "health centres" OR neighbourhood NEAR/2 "health centres" OR  CHCs OR  CHC OR health* NEAR/2 clinic* OR health* NEAR/2 service* OR primary NEXT healthcare OR primary NEXT "health care" OR primary NEXT care OR patient-centered NEXT care OR "patient centered" NEXT care OR patient-focused NEXT care OR "patient focused" NEXT care OR patient-centred NEXT care OR "patient centred" NEXT care OR hospital* NEAR/3 care OR hospital* NEAR/3 service* OR inpatient* NEAR/3 care OR inpatient* NEAR/3 service* OR in-patient* NEAR/3 care OR in-patient* NEAR/3 service* OR outpatient* NEAR/3 care OR outpatient* NEAR/3 service*OR  out-patient* NEAR/3 care OR out-patient* NEAR/3 service* OR ambulatory NEXT care OR urgent NEXT care OR clinic NEXT visit* OR  safety-net OR safety NEXT net OR  "Federally Qualified Health Center" OR  FQHC OR emergency NEXT department* OR emergency NEXT room* OR emergency NEXT ward*

#8 #1 OR #2 OR #3 OR #4 OR #5 OR #6 OR #7

#9 [mh "Social Determinants of Health"]

#10 [mh "Educational Status"]

#11 [mh ^"Employment"]

#12 [mh ^"Unemployment"]

#13 [mh ^"Poverty"]

#14 [mh "Food Supply"]

#15 [mh "Housing"]

#16 [mh "Public Assistance"]

#17 [mh "Social Welfare"]

#18 [mh "Insurance, Health"]

#19 [mh "Domestic Violence"]

#20 [mh "Transportation"]

#21 [mh "Interpersonal Relations"]

#22 [mh "Social Isolation"]

#23 social NEXT determinants* OR SODH OR SDH OR unmet NEXT need* OR under-met NEXT need* OR social NEXT need* OR economic NEXT need* OR education* NEAR/2 status OR education* NEAR/2 achievement* OR employ* OR unemploy* OR career* OR occupation* OR poverty OR income OR food NEXT supply OR food NEXT assistance OR food NEXT securit* OR food NEXT insecurit* OR housing OR homeless* OR rent* OR public NEXT assistance OR social NEXT welfare OR social NEXT service OR Medicare OR Medicaid OR childcare OR child NEXT care* OR day NEXT care* or daycare* OR utility NEXT assistance OR medical NEXT legal OR health NEXT insurance OR domestic NEXT violence OR family NEXT violence OR child NEAR/2 abuse OR elder NEXT abuse OR spous* NEXT abuse OR transport* OR commute OR commuting OR interpersonal NEXT safety OR social NEAR/2 interact* OR social NEAR/2 relationship OR social NEAR/2 isolat*

#24 #9 OR #10 OR #11 OR #12 OR #13 OR #14 OR #15 OR #16 OR #17 OR #18 OR #19 OR #20 OR #21 OR #22 OR #23

#25 [mh "Electronic Health Records"]

#26 electronic NEAR/2 medical record OR electronic NEAR/2 medical records OR electronic NEAR/2 health record OR electronic NEAR/2 health records OR personal NEAR/2 medical record OR personal NEAR/2 medical records OR personal NEAR/2 health record OR personal NEAR/2 health records OR medical NEAR/2 record* OR medical NEAR/2 health record OR medical NEAR/2 health records OR EHR* OR EMR* OR PHR*

#27 #25 OR #26

#28 [mh "Mass Screening"]

#29 screen* OR social NEXT history OR social NEXT risk* assessment OR social NEXT risk* assessments OR  "social need identification" OR  "social determinant assessment" OR  "social determinant screening" OR  "social determinant evaluation" OR  "social factor assessment" OR  "social factor screening" OR  "social determinant identification" OR socioeconomic NEXT screening

#30 #28 OR #29

#31 #8 AND #24 AND #27 AND #30 with Publication Year from 2015 to 2024, in Trials

**Supplementary file B: Full table of Summary of Approaches and Interventions for Addressing Social Determinants of Health: Impacts on Health Services Utilization and Costs**

| **Author (year)** | **Approach for screening for social determinants of health screened** | **Intervention to address the social determinants of health** | **Impact on health services utilization?** | **Health services utilization outcomes** | **Impact on healthcare costs?** | **Healthcare costs outcomes** |
| --- | --- | --- | --- | --- | --- | --- |
| Berkowitz et al. (2018) | Physician assessed nutritional risk, including food scarcity | Two meal programs delivered to participants’ homes, including five days of lunches, dinners, and snacks: 1) medically-tailored meal (MTM) customized to participants medical needs by a registered dietician, delivered weekly; 2) Meals on Wheels-type non-tailored food (NTF) without tailoring to medical needs, usually delivered daily | Yes, fewer ED visits and inpatient admissions | MTM program participants had fewer emergency department visits (Incidence Rate Ratio [IRR] 0.30; 95%CI 0.20 to 0.45) than matched non-participants, as did NTF program participants (IRR 0.56; 95%CI 0.47 to 0.68). MTM program participants also had fewer inpatient admissions (IRR 0.48; 95%CI 0.26 to 0.90). | Yes, lower medical expenditure | MTM program participants had lower medical expenditure (difference -$572, 95% CI -$933 to -$210). NTF program participation was associated with lower medical expenditure (difference -$159, 95%CI -$310 to -$8). |
| Bradley et al. (2018) | Student teams assessed patient at intake, on each visit, after 12 visits and at discharge, including demographics, personal characteristics, social determinants of health and utilization. A series of multiple choice questions addressed social determinants including on housing, health insurance, transportation, employment, safety/security, (e.g. “Where do you sleep?”) Questions were constructed to assess stability, e.g. “Has your housing situation changed in the last six months?” and “What is your source of monthly income?” Students documented assessments, into weekly chart notes and summaries. | Interprofessional Care Access Network (I-CAN): Student teams developed partnerships with clients to assist with navigating healthcare; accessing primary, specialty, and dental care; scheduling and coaching for appointments; providing reminders; arranging transportation to improve follow through; and attending appointments to support communication with the provider. | Yes, decrease in ED visits, emergency medical services and hospitalization, improved access to primary care | For 38 participants, substantial reductions compared to the 6 months before I-CAN in the aggregate number of emergency department visits (37 vs. 10), emergency medical service calls (25 vs. 8), and hospitalization (12 vs. 3). Self-reported access to primary care improved (49% to 63%). | Yes, lower costs for ED visits, hospitalization, and emergency service calls. | Estimated cost savings for the 38 clients, based on minimal estimated costs for number of ED visits, emergency medical service calls and hospitalization alone, were over $224,000 ($5,894 each). |
| Bronstein et al. (2015) | An individualized needs assessment conducted by Master of Social Work interns during the three primary points of intervention, identifying medication concerns, transportation issues, home care needs, home safety concerns, and behavioral barriers to follow-up care and activities. No tool was mentioned. | Post-discharge follow-up care coordination by Master of Social Work interns, including one or two phone calls (3 to 5 days and 21 days post discharge) and a home-visit (7-14 days). Interns empowered patients to find solutions, refer to appropriate resources, address concerns and ensure timely follow up with primary care provider. | Yes, reduced likelihood of readmission to hospital | Improve likelihood of not being readmitted by 22% (RR = 1.222; 95% CI = 1.0631.405). Highly significant risk improvement (χ2= 8.99; p= .003) | Not reported | Not reported |
| Capp et al. (2017) | Interview by a liaison from a community advocacy organization as part of the initial assessment about their experience with the healthcare system and needs (e.g., housing, insurance or disability benefits, refugee services, access to transportation, coordinating care and filling prescriptions) | Bridges to care (B2C) provides intensive medical, behavioral health, and social care coordination services, with up to eight home visits within sixty days of an ED visit or hospital discharge from a team comprising a primary care provider, care coordinator, health coach, behavioral health evaluator, and community health worker. Care is thereafter transferred to a patient centered medical home. | Yes, decrease in ED visits and hospitalizations and increase in primary care visits | Six months post intervention, significantly fewer ED visits (mean difference: 0.821, p< 0.05; a reduction of 27.9%) and hospitalizations (mean difference: 0.270, p<0.1; a reduction of 15.5%) and significantly more primary care visits (mean difference: 1.307, p< 0.05; an increase of 114.0%), compared to patients in the control group. | Not reported | Not reported |
| DeLaVega et al. (2022) | Patients were screened at the clinic every six months. The screening tool for health-related social needs included 8 domains of health-related social needs: housing insecurity, food insecurity, trouble paying for medications, trouble paying for transportation to medical appointments, trouble paying for utilities, need for employment, need for education, and difficulty taking care of children or other family members | At the end of the screening, a medical assistant asked patients if they wanted help connecting to resources. Patients who requested resources received printed information. Clinicians could also refer patients to a clinic-based patient navigator. Patients in the intervention group received enhanced pharmacy care with an additional focus on medication needs and health-related social needs, patient motivation, support to connect with resources, patient education, assistance with appointment scheduling and reminders | Yes, decrease in ED visits and inpatient hospital admissions for both groups. | Both groups experienced a decrease in ED visits and hospital admissions after 12-months, 129 (70.9%) vs 118 (64.8%) for screening and resources list, and 136 (74.7%) vs 110 (60.4%) for enhanced pharmacy. No significant difference between the two groups. | Not reported | Not reported |
| Finkelstein et al. (2020) | Screening for medical and social complexity (electronic medical record and written notes), including difficulty accessing services, lack of social support and homelessness. No tool mentioned. | The Camden Core Model is a post-discharge program with clinical and social components, including home visits, scheduling and accompanying patients to primary and specialty care visits, support navigating medical and social services (e.g., housing support, social support, social security). The team includes nurses, social workers, licensed practical nurses, community health workers, and health coaches. | No, no significant difference between intervention and control group in 180-day hospital readmissions rates | The 180-day readmission rate was 62.3% in the intervention group and 61.7% in the control group. The adjusted between-group difference was not significant (0.82 percentage points; 95% confidence interval, 5.97 to 7.61). | Not reported | Not reported |
| Gupta et al. (2023) | Social determinants of health screener entered into the EMR: 13-item questionnaire developed from validated and/or commonly used questions (e.g., hunger vital signs, food insecurity, housing, utilities, transportation, financial stability, violence/abuse, language, education, health literacy, social connectedness) | EMR-entered responses automatically triggered patients receiving a digital prescription for social determinants of health-related referrals | Yes, decrease in primary care visits | Patients receiving increasing volume of referrals was significantly associated with fewer primary care visits (−0.071, P = 0.002), but comorbidities moderated this effect. | Not reported | Not reported |
| Haggerty et al. (2023) | Screening for area material and social deprivation based on postal code and discussion on potential barriers with volunteer navigator (e.g., transportation) | Volunteer navigator outreach program: a single call offering a welcome service to patients assigned to a new family doctor at the clinic, offering information about consequence of not attending the first visit, documents required, logistical information about how to travel to the clinic, and preparation tips addressing identified barriers. Finally, they emailed brochures with information about the clinic and how to prepare for medical visits. | Yes, positive first visit with new primary care provider, increase in number of primary care visits | Post intervention increase in patients’ abilities to seek, reach and engage with care and helped them attach successfully to newly-assigned family doctors. Increase in primary care visits (2.00 at 3 months post intervention, p=0.04 vs. 1.2 in last 6 months at baseline). Decrease in ED use not significant | Not applicable |  |
| Heisler et al. (2022) | A comprehensive social needs assessment was done by a community health worker and summarized in a brief encounter form. No tool or domains mentioned. | Community health worker program: outreach by phone or in person to conduct an initial health, behavioral and social needs assessment, develop an action plan and link participants to services. | Yes, lower ED visits | Lower adjusted ratios of ED visits (adjusted rate ratio=0.96; p<.01) over 12-months, compared to the usual-care group. | Mixed, lower ED visit costs, higher ambulatory care costs, no difference in inpatient or total costs | Lower adjusted ratios of ED visit costs (adjusted rate ratio=0.96; p<.01).), but higher adjusted ratios of ambulatory care costs (adjusted ratio ratio=1.15; p<.01). No differences in inpatient or total costs compared with the usual-care group. |
| Henschen et al. (2022) | In-depth psychosocial assessment (e.g., housing, access to food and medication, transportation). No tool mentioned. | Complex High Admission Management Program (CHAMP): interdisciplinary team of social work, physicians, and pharmacists. used comprehensive care planning and inpatient, outpatient, and community visits to address both medical and social needs (e.g., connect patients to existing community resources, housing support, disability paperwork, access to food and medication, transportation). | No, increase in inpatient 30-day readmissions, no difference in hospital admissions, total hospital days ED visits and outpatient clinic visits. | After 180 days, CHAMP patients had more inpatient 30-day readmissions [CHAMP incidence rate 1.3 (95% CI 0.91.8) vs. control 0.8 (95% CI 0.51.1), p=0.04], though both groups had fewer readmissions compared to 180 days prior to enrollment. No differences in hospital admissions, total hospital days ED visits and outpatient clinic visits. | Not reported | Not reported |
| Kangovi et al. (2017) | Community health workers used a semi structured interview guide to understand social and behavioral determinants of health (e.g., food insecurity, housing instability, drug and alcohol use, social support) | Individualized Management for Patient-Centered Targets (IMPaCT) intervention delivered by community health workers providing tailored coaching, social support, advocacy, and navigation, including goal-setting, 6 months of tailored support (e.g., accompany to a food pantry), and connection with long-term support. Communicated with primary care provider about action plan. | Yes, lower hospitalizations (but not significant) | At 6-months, 16% of intervention group were hospitalized vs. 17.8% in the usual care group (P= .68). By 1 year, 23.3% of patients in the CHW arm were hospitalized versus 31.6%in the goal-setting arm (P = .11). At 1 year, there were 68 total hospitalizations (278 hospital days) in the CHW arm versus 98 (414 hospital days) in the goal-setting arm (P = .17). | Not reported | Not reported |
| Kangovi et al. (2018) | Community health worker conducted semi structured interview (open-ended) to assess socioeconomic determinants of health (e.g., trauma, food insecurity, housing instability, drug and alcohol use, or family stress). | Individualized Management for Patient-Centered Targets (IMPaCT) intervention delivered by community health workers including goal-setting, 6 months of tailored support, and connection with long-term support (e.g., attend social events, apply for social services, inform provider when patients cannot afford prescribed medication). | Yes, lower mean number of repeat hospitalizations and 30-day readmission (significant); fewer total days spent in hospital, shorter length of stay, fewer hospitalizations (not significant) | At 6 months, fewer total days spent in hospital for intervention group (155 days vs 345 days; absolute event rate reduction, 69%) and 9 months (300 days vs 471 days; absolute event rate reduction, 65%). Shorter average length of stay (difference, 3.1 days; 95% CI, 6.33 to 0.22; P = .06) and a lower mean number of hospitalizations (difference, 0.3; 95% CI, 0.6 to 0.0; P = .07) among hospitalized patients. Lower odds of repeat hospitalizations (OR, 0.4; 95% CI, 0.2-0.9; RD, 0.24; P=.02), including 30-day readmissions (OR, 0.3; 95% CI, 0.1-0.9; RD, 0.17; P=.04). | Not reported | Not reported |
| Kangovi et al. (2020) | Community health workers used a semi structured interview guide to understand social and behavioral determinants of health (e.g., food insecurity, housing instability, drug and alcohol use, social support). | Individualized Management for Patient-Centered Targets (IMPaCT) intervention delivered by community health workers providing tailored coaching, social support, advocacy, and navigation, including goal-setting, 6 months of tailored support (e.g., accompany to food pantry), and connection with long-term support. | Not reported | Not reported | Yes, every dollar invested in the intervention would return $2.47 within a year | Total savings for Medicaid $1,401,307.99. This savings divided by program expenses ($567,950.82) yielded a return of $2.47 for every dollar invested, realized within a single fiscal year. |
| Kenyon et al. (2016) | Collection of demographic data and systematically assessed social risk factors were part of midwife routine antenatal booking information (e.g. housing, teen parent, smoking, difficulty with English, benefit problem, UK resident for under a year, past or present mental illness, no social support, post code deprivation quintile, ethnicity, receipt of social services, drug or alcohol misuse in household, domestic abuse) | Pregnancy Outreach Worker service: provide individual support and case management for the women including home visiting from enrollment to 6 weeks after birth (e.g., encourage appointment attendance, make healthy choices, provider social/emotional support, manage benefits, housing difficulties and mental health problems). | No, no difference antenatal attendance, routine child assessment or primary immunization uptake. | Antenatal attendance: No difference between groups, either for all women or for women with two or more social risk factors (10.1 vs 10.1 (mean difference; MD) 0.00, 95% CI (95% CI 0.37 to 0.37)). Routine child assessment attendance and primary immunization uptake: not difference between groups | Not reported | Not reported |
| Kitzman et al. (2022) | Data on socioeconomics (based on zip code), gender, ethnicity, race, health insurance status). No tool or approach mentioned. | Baylor Scott & White Health and Wellness Center: one group received only primary care medical home services with “wrap-around” services (e.g., social worker, pharmacist, nutritionists, community health worker). In addition, the second group also received population health service such wellness, prevention or social determinants of health programs (e.g. access to affordable produce, support program) | Yes, decrease in ED and inpatient visits | Overall, 14% reduction in ED visits (p=.003) and 43% fewer inpatient visits (p<.0001) in year after enrollment, but moderated by chronic disease risk factors. | Yes, lower overall ED costs and inpatient costs, with lower ED costs among intervention group | Overall, 23.2% reduction in ED costs (p=.0007) and 49.5% reduction in inpatient costs (p<.0001). Participants in primary care medical home and population health service group had 37% lower ED costs than participants in the primary care medical home only (P=.01). |
| Liss et al. (2019) | A study programmer queried health system databases to collect data on patients sociodemographic characteristics and a post-discharge survey | Transitional care included a scheduled post-discharge appointment, where a multidisciplinary team comprehensively assessed patients medical and psychosocial needs, addressed modifiable barriers, and subsequent linkage to a new primary care source. | Yes, lower inpatient admissions at 90 and 180 days | Transitional care patients had 37% and 35% lower probability of any inpatient admission over 90 days (RR0.63; 95% CI 0.430.91) and 180 days (RR 0.65; 95% CI 0.470.89) and 42% fewer inpatient admissions over 180 days (incidence rate ratio 0.58; 95% CI 0.370.90). No significant difference between arms in the 90-day probability of death or additional hospital encounters (relative risk [RR] 0.89; 0.91; 95% confidence interval [CI] 0.741.13). | Not reported | Not reported |
| Losonczy et al. (2017) | Undergraduate volunteers screened patients for social, economic, environmental and legal needs and asked them to prioritize their top 3 (e.g., intimate partner violence, need for help with care of dependents, violence in school or community, housing conditions, education, transportation, income, ability to pay bills, immigration, asylum claims, legal needs, language needs, access to medical care). | Highland Health Advocates is an ED-based help desk and medical-legal partnership, where undergraduate volunteers help patients navigate public resources, provide onsite legal and social work referrals in English or Spanish and connect them to a medical home | Mixed, increase in having a medical home and doctor, no difference in ED utilization | At 1 month, increase in linkage with medical home in intervention group (92% vs 76%). At 6 months, more subjects in the intervention group had a doctor (93% v 69%). No difference was found in ED utilization. | Not reported | Not reported |
| Moreno et al. (2021) | Standardized comprehensive assessment to identify social needs (e.g. activities of daily living, health plan benefits, access to community resources, caregiver support, visual or hearing needs, culturally restricted treatments, barriers of access, transportation or financial, psychosocial factors, patients’ strengths and barriers as identified by provider) | Home-based social intervention delivered by a social worker and community health worker including initial assessment and plan, connection to social services supports in primary care and care coordination over 6 months (e.g., community referrals for food or housing, financial barriers, caregiver support) and communication with primary care provider | Yes, decrease in acute hospitalization and ED visits | After 12 months, pre/post reductions acute hospitalizations (Mean -0.66, IRR=.82, p=.006) and ED visits (Mean -0.57, IRR=.67, p=.003), compared to matched comparators | Not reported | Not reported |
| Onwuanyi et al. (2020) | Population health tool (Healthy Planet) used in the electronic medical record system to collect comprehensive social and behavioral details (e.g. financial barriers to medication) | Specific interventions related to identified social determinants of health (no details reported) in addition to usual medical care | Yes, reduction in 30-day readmissions and shorter length of stay | Less likely to be readmitted in 30 days, had shorter stay in the hospital and lower mortality (no statistics reported) | Not reported | Not reported |
| OToole et al. (2016) | Comprehensive assessment of needs (e.g., homelessness or high risk of homelessness, legal needs, access to care, food security, hygiene, clothing, employment, education, disability, transportation, social support). No tool or approach mentioned. | Homeless Patient Aligned Care Team (H-PACT) : enhanced, low-threshold access to care with open-access, walk-in capacity, flexible scheduling, clinical outreach to homeless people on streets, in shelters, and in community locations (e.g., soup kitchens, community resource centers); integrated services (i.e., mental health services and primary care services; sustenance needs (e.g., food or food vouchers, hygiene kits, clothes, bus passes, other transportation assistance) | Yes, reduced ED visits and hospitalizations | After 6 months, 19.0% reduction in ED use and a 34.7% reduction in hospitalizations. | Not reported | Not reported |
| Pantell et al. (2020) | Navigators administered baseline household social risk questionnaire: food security, problems paying utility bills, employment, housing, unhealthy environment, paying medical bills, health insurance, income support programs, lack of primary care physician, disability, access to mental health care, pregnancy-related benefits) | Navigator intervention: help caregivers resolve social needs, contact with families bi-weekly for up to 3 months, including clinic, government, or community resources targeted specifically to the social barriers that had been endorsed and prioritized by the caregiver. Control group: list of written resources not tailored to needs | Mixed, decrease in risk of hospitalization, no difference in ED visits. | After 1 year, there was no difference in risk of an emergency department visit between the 2 groups. Children enrolled in the in-person navigator group had a decreased risk of hospitalization within 12 months (hazard ratio, 0.59; 05% CI, 0.38-0.94; P = .03), making them 69% less likely to be hospitalized. | Not reported | Not reported |
| Pruitt et al. (2018) | Self-reported social needs (e.g. medical transportation, utility payments, food security, vision services, financial assistance, medication assistance, general transportation, housing support, rent assistance) | WellCare’s HealthConnections: Call center-based social service referral program to address social needs, such as homelessness, transportation barriers, and food insecurity | Not reported | Not reported | Yes, decrease overall expenditure | In the second year, decrease in mean expenditures for the group of participants who reported all their social needs met was $2443 (10%) greater than the group who reported none of their social needs met. |
| Roth et al. (2023) | Validated questions embedded into existing clinical workflows to systematic screening by primary care team (e.g., food, housing, utilities, transportation. | Providence Diabetes Collective Impact Initiative (DCII) included outreach, standardized protocols, diabetes self-management education, social needs screening, referral to a community resource desk (on-site resource staffed by community social service agent), and social needs support (e.g., transportation) | Mixed, increase in virtual primary care visits, no difference in primary care, ED, or inpatient utilization | Increase in the average number of virtual primary care visits of 0.35 per member, per year (p<0.001) compared to controls. No significant differences in the likelihood of an inpatient or emergency department, primary care, inpatient, and emergency department utilization. | Not reported | Not reported |
| Rucker et al. (2023) | BearSCREEN (Survey of Comprehensive Risk Evaluation and Emerging Needs), a 13-item computerized survey completed via tablet using REDCap software (e.g. literacy, housing, education, food security, risk for human trafficking, sexual health, safety practices, access to care, substance abuse, immigration, intimate partner violence, legal concerns, mental health needs) | All patients received a general list of community resources for all 13 social domains. Intervention group: received in-person, risk-tailored social needs navigation by a social navigator (study coordinator, medical student, or public health student) and patient co-developed a customized plan to access services for the reported social needs. | No, no difference in ED visits | After 12 months, no difference in number of subsequent ED visits for any visit type (46.5% vs. 46.2%; adjusted odds ratio [aOR] 1.0 [95% confidence interval {CI}: 0.7, 1.5]), or an SSV (17.7% vs. 21.5%; aOR 0.7 [95% CI: 0.4, 1.2]) | Not reported | Not reported |
| Schickedanz et al. (2019) | Phone screening: 14-question social needs questionnaire, follow by full assessment (10-15 minutes) if at least 1 social need identified (e.g., health literacy, education, financial insecurity, food insecurity, healthy food, housing, housing conditions, employment, child-related resources, transport, caregiver support, social isolation) | Health leads: A telephonic social needs screening, navigation, and referral program by Health Leads (at least a bachelor’s degree, assisted by social workers) Social resources information was provided immediately over the phone or during a follow-up call. | Yes, decrease in ED, outpatient, and inpatient visits | After 1 year, 2.2% decrease in total visit count for intervention group (outpatient, ED and inpatient) (95% CI 4.5%, 0.1%; p =0.058). ED visit count decreased by 0.06, inpatient hospitalizations by 0.08 and ambulatory visits by 0.03 for intervention patients compared with controls. Effect was larger in all low socioeconomic group receiving the intervention (7.0 to 12.1% decrease). | Not reported | Not reported |
| Schumacher et al. (2017) | Health literacy screening (Rapid Estimate of Adult Literacy in Medicine) | The ED-to-home intervention (modeled on Transitions Intervention): trained coaches from community area agencies helped patients schedule follow-up doctor visits; recognize disease worsening; reconcile medications; and communicate with providers, including home visit (3 days after discharge), 3 calls (over 1 month). | No, no difference in primary care doctor visits | No difference in post ED follow-up doctor visits | Not reported | Not reported |
| Sege et al. (2015) | Questions to determined upstream factors such as food, housing, utilities, hardship, family legal needs. No tool or approach mentioned. | Developmental Understanding and Legal Collaboration for Everyone (DULCE): Family Specialist provided support and assisted with access to resources (e.g. food stamps, utilities assistance, housing voucher) until the 6-month routine health care visit, including collaborative routine visits with the family and the medical provider, home visits; and contact by telephone, e-mail, text, or in person. | Yes, increase in routine preventive care, decreased ED visits | By age 1, intervention infants were more likely to have 5 or more routine preventive care visits (78% vs 67%, P = .01), 93% (vs. 86%) of intervention families continued to receive primary care at the study site (P = .056). Likelihood of ED visits was significantly lower in intervention group at 6 months (36.5% vs 49.7%, P = .021), but not significant at 12 months (59.3% vs 65.0%, P = .40). Total ED visits was lower at 6 months (P = .023) but not at months (P = .08). | Not reported | Not reported |
| Smith et al. (2021) | Electronic medical record screening for frequent ED use, homelessness, and chronic pain. | Frequent User System Engagement (FUSE): coordinates multidisciplinary care (nurse, social worker, peer support, mental health), stable housing through dedicated 10 independent appartements and referrals to community resources for housing, employment or other support. | Yes, decrease in ED visits, increase in primary care provider visits and diagnostic tests | After 18 months, significant decrease in ED visits (χ2 (5, n = 11) = 13.47, P = .02) a, increase in primary care provider visits (χ2 (5, n = 11) = 30.12, P < .01), and increase in number of diagnostic tests (χ2 (5, n = 11) = 27.37, P < .01) | Mixed, no difference for ED and total costs, increase for diagnostic tests | Significant increase in costs of diagnostic tests (χ2 (5, n = 11) = 16.08, P < .01). Differences in ED costs (χ2 (5, n = 11) = 10.44, P = .06) and total costs (χ2 (5, n = 11) = 8.34, P = .14) were not significant |
| Vasan et al. (2020) | Community health workers used a semi structured interview guide to understand social and behavioral determinants of health such as food insecurity, housing instability, drug and alcohol use, social support) | Individualized Management for Patient-Centered Targets (IMPaCT): community health workers provide goal setting, tailored social support, health behavior coaching, connection long-term support (resources, and health system navigation). | Yes, decrease in hospitalizations and length of stay | Over 9398 observed patient months (1 to 12 months), total number of hospital days per patient in the intervention group was 34% lower (IRR) 0.66, P < .0001)). This reduction was driven by fewer hospitalizations per patient (0.27 vs 0.34, P < .0001) and shorter mean length of stay (4.72 vs 5.57 days, P = .03). Rates of hospitalization also decreased outside patients' primary health system (18.8 percent vs 34.8 percent, P = .0023). | Not reported | Not reported |
| Vest et al. (2018) | Based on provider assessment of social needs or proactive review of records of patients with scheduled appointments. No tool or domains mentioned. | Eskenazi Health Wraparound services (e.g. social worker, dietician, behavioral health): col-located at outpatient clinics that directly address social determinants of health | Yes, decrease in ED visits and hospitalization | After 1 year, 7% decrease in expected number of hospitalizations and 5% decrease in ED visits. Number of nonemergent ED visits was lower, but not significant. | Yes, decrease in costs from hospitalizations | Estimated cost savings from potentially avoided hospitalizations alone was $1.4 to $ 2.4 million annually ($76-$131 per person) |
| Wallace et al. (2020) | ED registration staff collected data a 10-item social needs screening tool (adapted from Health Leads) in Spanish or English, adapted for 5th grade literacy and long-term risk over 12 months (e.g., transportation, ability to pay for care or medication, ability to pay for food, clothing or furniture, utilities payments, mortgage or rent payments, housing security, unemployment, access to child or elder care) | A direct (electronic) referral for patients who agreed to be referred to 2-1-1 service: free, comprehensive list of contact information for local providers who address common social needs (e.g. housing, utilities, food assistance, transportation, legal, mental health and addiction, insurance, employment, education, domestic violence) | No, no difference in hospitalization, increase in ED use | After 3 months, patients with at least 1 social need had a significant increase in ED use (1.07 before vs 1.36 after, P = .03) while patients with no needs had an increase in primary care visits (0.24 before vs 0.56 after, P = .03). ED visits increased among those who received follow-up and referrals from 2-1-1 (1.97 before vs 2.56 after, P = .006). No differences in hospitalizations. | Not reported | Not reported |
| Weerahandi et al. (2015) | Comprehensive psychosocial assessment during the index hospitalization. No tool or domains mentioned. | Preventable admissions care team (PACT): social work transitional care model with tailored interventions, including phone calls, home visits, accompanying patients to appointments, follow-up for 35 days after hospital discharge | Yes, decrease in hospital readmissions | Compared to controls, 30-day readmission rate decreased by 34% (p = <0.001), 60-day hospitalization rate decreased by 22% (p = 0.004); 90-day hospitalization rate decreased by 19% (p = 0.006). | Yes, lower overall costs | Inpatient costs 30 days post-index were $2.7 million for PACT patients and $3.6 million for controls. |
| Woodhead et al. (2017) | Financial strain identified by practice staff or self-identified by patients (e.g., education, employment, tenure, income, problem paying for housing). No tool mentioned. | Primary care co-located debt and welfare advice: citizen advice service providing specialist in-depth advice at general practices, primarily about welfare benefits and debts. | No, no changes in primary care visits | No changes in 3-months consultation rates with general practitioner | Yes, financial benefit to recipient returns on investment | Per capita, advice recipients received £15 per £1 of funder investment. |
| Wu et al. (2019) | Screening for high risk through a predictive model or risk identified by their provider. No tool or domains mentioned. | Baltimore Community-Based Organizations Neighborhood Network: Enhancing Capacity Together (CONNECT): online tool to help refer clients to community resources, meet-and-greet sessions between community-based organization staff and healthcare staff, and research assistants. | No, no difference in ED visits or hospital days | There was no significant effect of the intervention on healthcare utilization outcomes, including ED visits and days spent in hospital | Not reported | Not reported |
| Xiang et al. (2019) | Thorough biopsychosocial needs assessment (e.g., health literacy, support, living alone, inadequate access to community long-term care, unstable or unsafe home environment, transportation). No tool mentioned. | Bridge Model for Super Utilizers (Bridge-SU): social work-based transitional care designed to address the medical and social needs of inpatient super utilizers (5+ hospital admissions per year), where social worker coordinators help address psychosocial factors, care coordination and case management. | Yes, decrease in hospital admissions, readmissions, and ED visits | After 12 months, significant reduction in the total number of hospital admissions (pre 5.76 (95% CI = 5.64–5.88) vs post 2.38 (95% CI = 2.17–2.59), nearly halved 30-day readmission rates (pre: 25.5% (95% CI = 24.7–26.4%) vs post 13.4% (95% CI = 12.0–14.8%), and number of emergency department visits (pre: average of 5.39 visits (95% CI = 5.09–5.68) vs. post 3.38 visits (95% CI = 2.78–3.98) | Yes, decrease in hospitalization costs | After 12 months, significant reductions in average hospital charges per episode by $14,150, and total hospital charges per person decreased by nearly $200,000. |

**Supplementary file C: MMAT Quality assessment**

| **Author (year)** | **High** | **low** | **Medium** | **Total** |
| --- | --- | --- | --- | --- |
|  |  |  |  |  |
| **Mixed methods** | 2 | 1 | 3 | 6 |
| Bradley 2018 |  |  | 1 | 1 |
| Haggerty 2023 |  | 1 |  | 1 |
| Onwuanyi 2020 | 1 |  |  | 1 |
| Schumacher 2017 |  |  | 1 | 1 |
| Vest 2018 |  |  | 1 | 1 |
| Wallace 2020 | 1 |  |  | 1 |
| **Quantitative descriptive** | | 2 |  | 2 |
| Gupta 2023 |  | 1 |  | 1 |
| OToole 2016 |  | 1 |  | 1 |
| **Quantitative non-randomized** | **1** | **4** | **8** | **13** |
| Berkowitz 2018 |  | 1 |  | 1 |
| Capp 2017 |  | 1 |  | 1 |
| DeLaVega 2022 |  | 1 |  | 1 |
| Kitzman 2022 |  | 1 |  | 1 |
| Losonczy 2017 | 1 |  |  | 1 |
| Moreno 2021 |  |  | 1 | 1 |
| Pruitt 2018 |  |  | 1 | 1 |
| Roth 2023 |  |  | 1 | 1 |
| Schickedanz 2019 |  |  | 1 | 1 |
| Smith 2021 |  |  | 1 | 1 |
| Weerahandi 2015 |  |  | 1 | 1 |
| Woodhead 2017 |  |  | 1 | 1 |
| Xiang 2019 |  |  | 1 | 1 |
| **Quantitative randomized controlled trials** | **1** | **7** | **6** | **14** |
| Bronstein 2015 |  |  | 1 | 1 |
| Finkelstein 2020 |  | 1 |  | 1 |
| Heisler 2022 |  |  | 1 | 1 |
| Henschen 2022 |  | 1 |  | 1 |
| Kangovi 2017 |  | 1 |  | 1 |
| Kangovi 2018 |  | 1 |  | 1 |
| Kangovi 2020 |  | 1 |  | 1 |
| Kenyon 2016 |  | 1 |  | 1 |
| Liss 2019 |  |  | 1 | 1 |
| Pantell 2020 |  |  | 1 | 1 |
| Rucker 2023 | 1 |  |  | 1 |
| Sege 2015 |  |  | 1 | 1 |
| Vasan 2020 |  |  | 1 | 1 |
| Wu 2019 |  | 1 |  | 1 |
| **Grand Total** | **4** | **14** | **17** | **35** |
